# A genetic risk score based on *BCL11A* and *HBS1L‐MYB* variants predicts clinical severity in Brazilian sickle cell anaemia patients

**DOI:** 10.1111/bjh.70489

**Published:** 2026-04-16

**Authors:** Gabriela S. Arcanjo, Alexsandro P. Silva, Madi V. Diniz, Igor F. Domingos, Diego A. Pereira‐Martins, Amanda B. Araújo, Talita S. S. França, Ana C. Anjos, Aderson S. Araujo, Edis Belini‐Junior, Sara T. O. Saad, Fernando F. Costa, Antonio R. Lucena‐Araujo, Marcos André C. Bezerra

**Affiliations:** ^1^ Genetics and Molecular Biology Postgraduate Program Federal University of Pernambuco Recife Pernambuco Brazil; ^2^ Hematology and Hemotherapy Center State University of Campinas Campinas São Paulo Brazil; ^3^ Cardiology Emergency Unit of Pernambuco University of Pernambuco Recife Pernambuco Brazil; ^4^ Department of Hematology, Cancer Research Centre Groningen University Medical Centre Groningen, University of Groningen Groningen The Netherlands; ^5^ Department of Internal Medicine Hematology and Hemotherapy Foundation of Pernambuco Recife Pernambuco Brazil; ^6^ Molecular Biology and Genetics Laboratory Federal University of Mato Grosso do Sul (UFMS) Três Lagoas MS Brazil

**Keywords:** fetal haemoglobin, haplotypes, HMIP, rs1427407, rs9399137, sickle cell disease

## Abstract

Individuals with sickle cell anaemia (SCA) exhibit significant clinical heterogeneity influenced by several factors, especially fetal haemoglobin (HbF) levels. Variations in adult HbF levels are partly explained by the co‐inheritance of genetic variants that regulate globin expression. In this study, we investigated the association of *BCL11A* rs4671393, rs1427407, rs11886868 and *HBS1L‐MYB* rs9399137 polymorphisms with HbF levels and clinical complications in a cohort of 409 adult Brazilian SCA patients. Our findings reveal that variant alleles of all four single‐nucleotide polymorphisms (SNPs) were significantly associated with higher HbF levels. Moreover, homozygosity for the major alleles was independently associated with higher risk and cumulative incidence of stroke, avascular necrosis, leg ulcers, priapism and acute chest syndrome. Haplotype analysis with *BCL11A* variants was also associated with HbF and the patient's phenotype. A genetic risk score (GRS) combining the risk genotypes was significantly associated with lower HbF levels (*p* < 0.0001) and increased complication risk (*p* < 0.0001). A model integrating the GRS with clinical variables demonstrated superior discriminatory performance (area under the curve (AUC): 0.72) compared to models based solely on clinical factors. In summary, this study underscores the clinical relevance of HbF‐related genetic variants and supports their integration into risk stratification and personalized management strategies for SCA.

## INTRODUCTION

In sickle cell anaemia (SCA), the severity of the clinical phenotype is influenced by multiple factors, with fetal haemoglobin (HbF) being one of the most critical determinants. Elevated HbF levels have been associated with reduced haemoglobin polymerization, decreased haemolysis and lower rates of vaso‐occlusive crises and organ damage in SCA.^1^, ^2^, ^3^ Consequently, HbF is a major protective factor in SCA, and its modulation remains a key therapeutic target for improving clinical outcomes in haemoglobinopathies.^4^

Several erythroid‐specific transcriptional regulators, primarily B‐cell lymphoma/leukaemia 11A (BCL11A), Krüppel‐like factor 1 (KLF1), and MYB proto‐oncogene, transcription factor (c‐Myb), mediate the repression of HbF expression after birth.^5^, ^6^ Inter‐individual variability in HbF levels in individuals with SCA is partially attributed to the co‐inheritance of genetic variants that influence the regulatory regions of these key globin regulators.^7^ Multiple genetic modifiers, including single‐nucleotide polymorphisms (SNPs) and quantitative trait loci (QTLs), have been identified as major determinants of HbF levels. Among these, the *XmnI*/*HBG2* (11p15.4) variant in the promoter region of the *HBG2* gene as well as QTLs in the *BCL11A* gene (2p16.1) and the intergenic region of *HBS1L‐MYB* (6q23.3) have been strongly associated with elevated HbF levels, improved clinical outcomes and therapeutic response in SCA.^8^, ^9^, ^10^, ^11^

BCL11A is a transcription factor that represses γ‐globin expression through direct binding to the *HBG* promoter and interactions with erythroid regulators.^6^, ^12^ Functionally relevant variants within a 14‐kb erythroid enhancer in intron 2 of *BCL11A* (including rs1427407, rs4671393 and rs11886868) alter chromatin organization and transcription factor binding, reducing *BCL11A* expression and leading to increased HbF levels.^13^, ^14^, ^15^ The *HBS1L‐MYB* locus also plays a central role in HbF regulation: *HBS1L* encodes a guanosine triphosphate‐binding protein,^8^ while *MYB* encodes the transcription factor c‐Myb, a key regulator of haematopoiesis and HbF expression.^16^, ^17^ Variants within the *HBS1L‐MYB* intergenic region (HMIP) reduce binding of key erythroid transcription factors and long‐range regulation of *MYB*, with multiple HbF‐associated QTLs identified in this region, particularly within the HMIP‐2 block (e.g. rs9399137).^15^, ^18^ Given the pivotal role of these genetic factors in modulating HbF expression, this study aimed to investigate the association of *BCL11A* and *HBS1L‐MYB* genetic variants with HbF levels and the clinical phenotype of Brazilian SCA patients.

## METHODS

### Patients

This study included 409 unrelated adult patients with SCA who were regularly followed at a single reference centre in north‐eastern Brazil. Haemoglobin S homozygosity was confirmed for all participants through cation‐exchange high‐performance liquid chromatography and restriction fragment length polymorphism analysis of a PCR‐amplified fragment of the beta gene using *DdeI* enzyme. Clinical and laboratory data were retrospectively obtained from patients' medical records (Table 1). Baseline laboratory parameters were assessed during treatment‐free periods for patients who had received clinical interventions, such as hydroxycarbamide (hydroxyurea (HU)) therapy or chronic blood transfusions.

**TABLE 1:** Baseline characteristics of SCA patients according to the disease status.

Characteristics of patients	All patients (*n* = 409)		Symptomatic SCA (*n* = 291)		SCA controls (*n* = 148)		*p*‐value**
	No.	%	No.	%	No.	%	
Gender							
Female	215	52.6	114	43.7	101	68.2	<0.001*
Male	194	47.4	147	56.3	47	31.8	
Age (years), median	34 (15)		34 (13)		34 (16)		0.657
VOCs/per year							
<3	193	47.5	107	41.5	86	58.1	0.005*
3–6	153	37.7	107	41.5	46	31.1	
>6	60	14.8	44	17.1	16	10.8	
HbF (%)	6.7 (6.8)		5.8 (5.6)		8.9 (9.3)		<0.0001*
RBC (×10^6^/μL)	2.53 (0.58)		2.51 (0.58)		2.59 (0.58)		0.047*
Hb (g/dL)	7.8 (1.4)		7.7 (1.4)		8.0 (1.4)		0.045*
Haematocrit (%)	23.4 (4.6)		23.1 (4.4)		24.1 (4.1)		0.103
Reticulocyte (%)	10 (7.0)		10.2 (7.2)		9.3 (7.2)		0.359
WBC (×10^9^/L)	12.1 (5.1)		12.5 (5.4)		11.2 (5.0)		0.167
Platelets (×10^9^/L)	425 (170)		422 (175)		422 (172)		0.715
TB (mg/dL)	2.8 (2.2)		3.0 (2.3)		2.6 (2.0)		0.087
IB (mg/dL)	2.1 (2.1)		2.2 (2.2)		2.0 (1.9)		0.067
LDH (U/L)	807 (601)		876 (737)		713 (455)		0.037*
*β* ^S^ haplotype							
CAR/CAR	222	57.4	142	57.0	80	58.0	
Non‐CAR/CAR	165	42.6	107	43.0	58	42.0	0.915
NA	22	—	12	—	10	—	
α‐thalassaemia (*α* ^−3.7kb^)							
Mutated	93	23.8	55	22.0	38	27.0	
Non‐mutated	298	76.2	195	78.0	103	73.0	0.296
NA	18	—	11	—	7	—	

*Note*: Laboratorial parameters are described as median (interquartile range (IQR)). Mutated alpha‐thalassaemia is defined by one or two deletional *α* genes.

Abbreviations: CAR, Central African Republic; Hb, haemoglobin; HbF, fetal haemoglobin; IB, indirect bilirubin; LDH, lactate dehydrogenase; NA, not available; RBC, red blood cell; SCA, sickle cell anaemia; TB, total bilirubin; VOC, vaso‐occlusive crisis; WBC, white blood cells.

* Statistically significant difference (*p* < 0.05).

** Fisher's exact or chi‐square tests for categorical variables. Mann–Whitney or Kruskal–Wallis tests for continuous variables.

Patients were classified into subgroups based on the presence of clinical complications, including stroke, avascular necrosis (AVN), leg ulcers (LUs), priapism and acute chest syndrome (ACS). Patients were also categorized according to the frequency of vaso‐occlusive crisis (VOCs) requiring hospitalization in the year preceding the end of the follow‐up period. Details of the patient's clinical characterization are described in the Supplemental Data. The control group consisted of SCA patients over 18 years of age who had not developed any of the five major complications by the time of study censure. The study was approved by the local research ethics board (protocol approval number: 42396621.0.0000.5208). Written informed consent was obtained from all participants before the study commenced.

### Molecular data

Peripheral blood samples were collected at a single time point during patient recruitment for DNA extraction.^19^ All patients were fully genotyped for *BCL11A* rs4671393, rs1427407, rs11886868 and *HBS1L‐MYB* rs9399137. Genotyping was performed using real‐time polymerase chain reaction (PCR) with TaqMan® probes (Applied Biosystems, Foster City, CA, USA). Details of the TaqMan probes and PCR conditions are described in the Supplemental Data. Additionally, β^S^‐globin gene cluster haplotypes and the co‐inheritance with ^−3.7Kb^alpha‐thalassaemia were determined as previously described.^20^, ^21^

### Statistical analyses

Descriptive statistics were used to summarize baseline characteristics. Genotype distributions were tested for Hardy–Weinberg equilibrium (HWE). Linkage disequilibrium (LD) and haplotype analyses of *BCL11A* SNPs were conducted using Haploview software (v. 4.2).^22^ Linear regression under an additive genetic model was used to assess the associations of *BCL11A* and *HBS1L‐MYB* SNPs and *BCL11A* haplotypes with HbF levels. Logistic regression was used to evaluate associations with clinical complications, with results expressed as odds ratios (ORs) and 95% confidence intervals (CIs). Time‐to‐event analyses were performed using Kaplan–Meier curves and Cox proportional hazard models to estimate cumulative risk. Multivariable models were adjusted for gender, age and the number of VOCs/year.

A genetic risk score (GRS) was constructed to capture the cumulative effect of risk alleles and *BCL11A* haplotype and evaluated for associations with HbF levels and clinical complications. Details of the statistical methods are described in the Supplemental Data. All statistical analyses were performed using SPSS Statistics 19.0 (IBM Corporation, Somers, NY, USA) and the R software (The CRAN project), with *p* < 0.05 considered statistically significant.

## RESULTS

### Patients' characterization

The cohort included 409 patients with SCA (median age 34 years), of whom 215 were females (52.6%). Overall, 261 patients (63.8%) developed at least one of the five clinical complications studied and were classified as symptomatic. Symptomatic patients were grouped for descriptive analyses with the control group. Baseline clinical and laboratory data are presented in Table 1. Patients with symptomatic SCA had a higher proportion of males (*p* < 0.001), more VOC episodes (*p* = 0.005), lower HbF levels (*p* < 0.0001), lower red blood cell (RBC) counts (*p* = 0.047) and haemoglobin (Hb) levels (*p* = 0.045) as well as higher lactate dehydrogenase levels (*p* = 0.037) compared with the control group. Baseline HbF levels were consistently lower in each complication group than in complication‐free patients (Table S1).

### *BCL11A* and *HBS1L* *‐* *MYB* genotypes, *BCL11A* haplotypes and fetal haemoglobin levels

**FIGURE 1:**
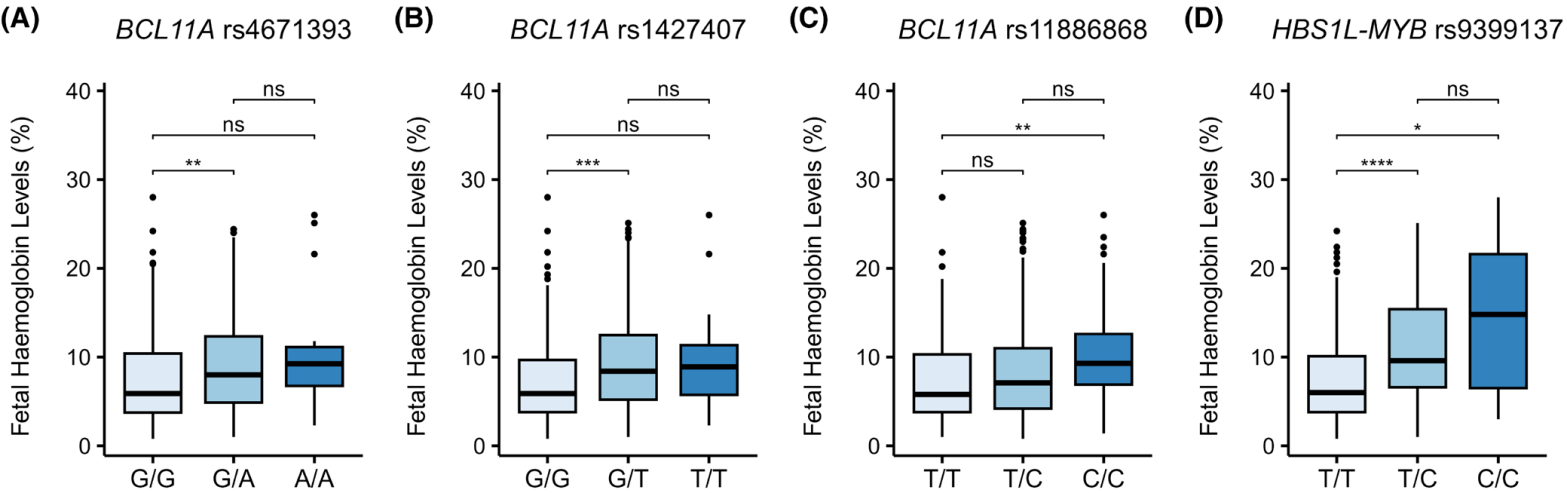
Association between fetal haemoglobin (HbF) levels and genetic variants in *BCL11A* and *HBS1L‐MYB*. Box plots depict HbF levels (%) in sickle cell anaemia patients stratified by genotype for four single nucleotide polymorphisms: (A) *BCL11A* rs4671393 [GG (*n* = 199), GA (*n* = 160), AA (*n* = 16)], (B) *BCL11A* rs1427407 [GG (*n* = 206), GT (*n* = 154), TT (*n* = 15)], (C) *BCL11A* rs11886868 [TT (*n* = 141), TC (*n* = 185), CC (*n* = 49)] and (D) *HBS1L‐MYB* rs9399137 [TT (*n* = 281), TC (*n* = 85), CC (*n* = 9)]. Only individuals with available HbF measurements were included. Statistical significance was assessed using the Kruskal–Wallis test followed by Dunn's multiple comparisons test. Significance levels are indicated as follows: *p* < 0.05, *p* < 0.01, *p* < 0.001, *p* < 0.0001; ns, not significant.

Kruskal–Wallis tests with Dunn's post hoc comparisons revealed that rs4671393, rs1427407, rs11886868 and rs9399137 were significantly associated with HbF levels. For rs4671393, median HbF values were 5.9% (GG), 8.0% (GA) and 9.25% (AA) (*p* = 0.002). rs1427407 showed median HbF levels of 5.9% (GG), 8.4% (GT) and 8.9% (TT) (*p* < 0.001). For rs11886868, medians were 5.8% (TT), 7.1% (TC) and 9.3% (CC) (*p* = 0.005). rs9399137 showed the strongest association (*p* < 0.0001), with median HbF values rising from 6.0% (TT) to 9.6% (TC) and 14.8% (CC) (Figure 1). Linear regression models showed that all minor alleles of these SNPs were associated with increased HbF levels. Among the *BCL11A* variants, rs1427407 exhibited the strongest effect (*β* = 0.233, *p* < 0.0001) and explained 7.7% of the variance in HbF levels. However, the *HBS1L‐MYB* variant rs9399137 demonstrated the most substantial effect (*β* = 0.356, *p* < 0.0001), accounting for 14.9% of HbF variance (Table 2).

**TABLE 2:** Linear regression analysis of *BCL11A* and *HBS1L‐MYB* SNPs with HbF levels under an additive allelic model.

SNP	Minor allele (HbF‐boosting allele)	MAF	*B*	*β*	*p*‐value	*R* ^2^ (variance explained (%))
*BCL11A*						
rs4671393	A	0.27	1.937	0.204	<0.0001	6.5%
rs1427407	T	0.26	2.233	0.233	<0.0001	7.7%
rs11886868	C	0.39	1.444	0.176	<0.001	5.4%
*HBS1L‐MYB*						
rs9399137	C	0.14	3.930	0.356	<0.0001	14.9%

*Note*: The effect size (standardized (*β*) and unstandardized (*B*) regression coefficients) represents the estimated increase in HbF per minor allele. The *R* ^2^ values indicate the percentage of variance in HbF explained by each SNP.

Abbreviations: HbF, fetal haemoglobin; MAF, minor allele frequency; SNP, single nucleotide polymorphism.

Haploview software indicated that all three *BCL11A* SNPs met the qualifications for LD analysis: SNPs with a genotype call rate of 100%, minor allele frequency >1%–2% and HWE without extreme deviation with *p* < 0.001.^23^ Pairwise LD analysis revealed that the SNPs are not completely redundant. *r* ^2^ values ranged from moderate (0.43–0.52) to strong (0.78). *D*′ values exceeded 0.89 for all SNP pairs. High LOD scores (range: 48.87–96.8) indicate strong evidence against independent segregation (Table S2). High *D*′ and LOD scores, along with *r* ^2^ values, support the use of the Solid Spine of LD for haplotype analysis. The method generated one block containing three *BCL11A* SNPs, resulting in the haplotypes GTG (*f* = 0.590), TCA (*f* = 0.241), GCG (*f* = 0.123), GCA (*f* = 0.022) and TTG (*f* = 0.013) (Figure S1).

To evaluate the association between *BCL11A* haplotypes and HbF levels, individuals were classified into three groups according to the presence or absence of the *BCL11A* haplotype carrying high‐HbF alleles (rs1427407‐T, rs11886868‐C and rs4671393‐A), defined as TCA−/TCA−, TCA−/TCA+ and TCA+/TCA+. Linear regression analysis revealed that each additional copy of the TCA+ haplotype was significantly associated with increased HbF levels (*β* = 0.224; *p* < 0.0001), accounting for 6.7% of the variance in HbF.

### *BCL11A* and *HBS1L* ‐ *MYB* genotypes, *BCL11A* haplotype and SCA clinical complications

Stroke risk was independently associated with homozygosity for the major allele for rs4671393 (OR = 2.11, *p* = 0.019), rs1427407 (OR = 1.93, *p* = 0.037) and rs9399137 (OR = 3.63, *p* = 0.002) (Table S3). Higher risk of AVN was also observed with major genotypes for rs4671393 (OR = 2.05, *p* = 0.027), rs1427407 (OR = 2.16, *p* = 0.018) and rs9399137 (OR = 2.80, *p* = 0.008) (Table S4).

Homozygosity for the major allele for rs4671393 (OR = 2.05, *p* = 0.027), rs1427407 (OR = 2.50, *p* = 0.003), rs11886868 (OR = 2.52, *p* = 0.009) and rs9399137 (OR = 2.42, *p* = 0.011) was independently associated with an increased risk of LUs (Table S5). Major homozygous genotypes for rs4671393 (OR = 2.29, *p* = 0.040) and rs1427407 (OR = 3.27, *p* = 0.004) were independently associated with an increased risk of priapism (Table S6). Finally, for ACS, major homozygous genotypes for rs4671393 (OR = 1.94, *p* = 0.029) and rs1427407 (OR = 2.07, *p* = 0.018) were independently associated with ACS risk (Table S7).

The association between the *BCL11A* TCA haplotype and clinical complications was evaluated using multiple binary logistic regressions. Under the additive model, carriers of the TCA− (non–HbF‐boosting) haplotype showed increased risk for LUs (OR = 2.01, *p* = 0.009), priapism (OR = 2.43, *p* = 0.024) and ACS (OR = 1.99, *p* = 0.018) (Table S8).

### Long‐term risk of SCA complications according to *BCL11A* and *HBS1L* ‐ *MYB* genotypes and *BCL11A* haplotype

**FIGURE 2:**
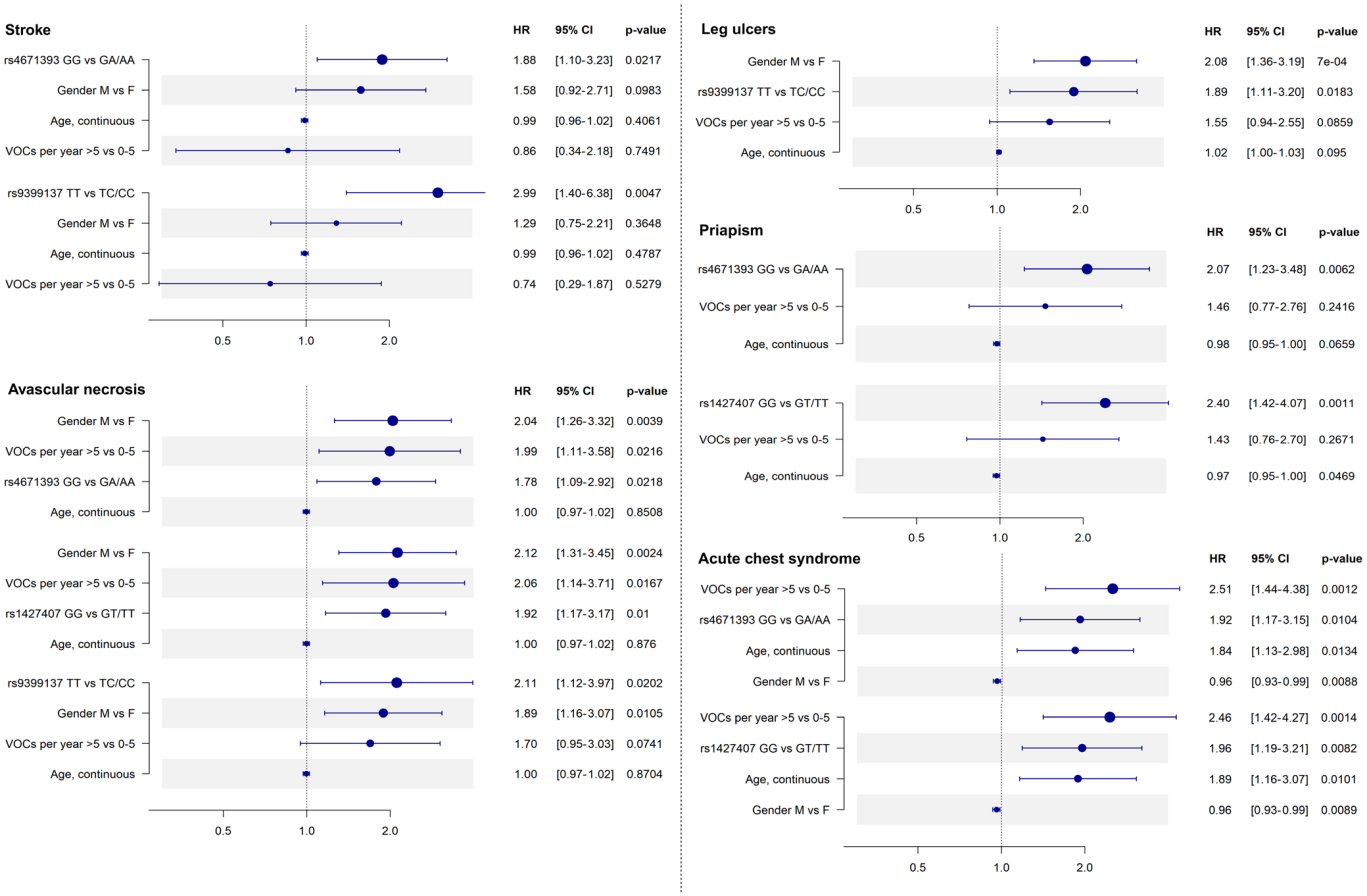
Forest plots of Cox proportional regression analysis for genetic and clinical predictors of sickle cell anaemia (SCA) complications. Analyses were performed for stroke, priapism, avascular necrosis, acute chest syndrome and leg ulcers. The evaluated genetic variants include *BCL11A* rs4671393, *BCL11A* rs1427407, *BCL11A* rs11886868 and *HBS1L‐MYB* rs9399137. Clinical covariates such as gender (male vs. female), age (continuous variable) and frequency of vaso‐occlusive crises (VOCs per year: >5 vs. 0–5) were included in the regression models. Hazards ratio (HRs) greater than 1 indicate increased risk, while HRs below 1 indicate a protective effect for the first category listed. Adjusted *p*‐values are presented.

To further explore the relationship between the SNPs and the cumulative incidence of complications, we conducted Kaplan–Meier survival analyses for each significant SNP identified in the logistic regression. The resulting curves (Figures S2 and S3) revealed genotype‐dependent differences in complication risk over time. Multivariable Cox proportional hazard models confirmed these findings, with several SNPs remaining significantly associated. Specifically, individuals carrying the major GG genotype of rs4671393 had a higher risk of stroke (Hazards ratio [HR] = 1.88, *p* = 0.021), AVN (HR = 1.78, *p* = 0.021), priapism (HR = 2.07, *p* = 0.006) and ACS (HR = 1.95, *p* = 0.014). Individuals with the GG genotype for rs1427407 exhibited an increased risk of AVN (HR = 1.92, *p* = 0.011), priapism (HR = 2.40, *p* = 0.001) and ACS (HR = 1.96, *p* = 0.008). Additionally, rs9399137 TT was significantly associated with an increased cumulative risk of stroke (HR = 2.99, *p* = 0.004), AVN (HR = 2.11, *p* = 0.020) and LUs (HR = 1.89, *p* = 0.018) (Figure 2).

Finally, in accordance with the logistic regressions, the *BCL11A* TCA− (non–HbF‐boosting) haplotype was independently associated with increased cumulative risk for LUs (HR = 1.46, *p* = 0.046), priapism (HR = 2.10, *p* = 0.006) and ACS (HR = 1.76, *p* = 0.018) (Table S8).

### *BCL11A* and *HBS1L* ‐ *MYB* genetic risk score

**FIGURE 3:**
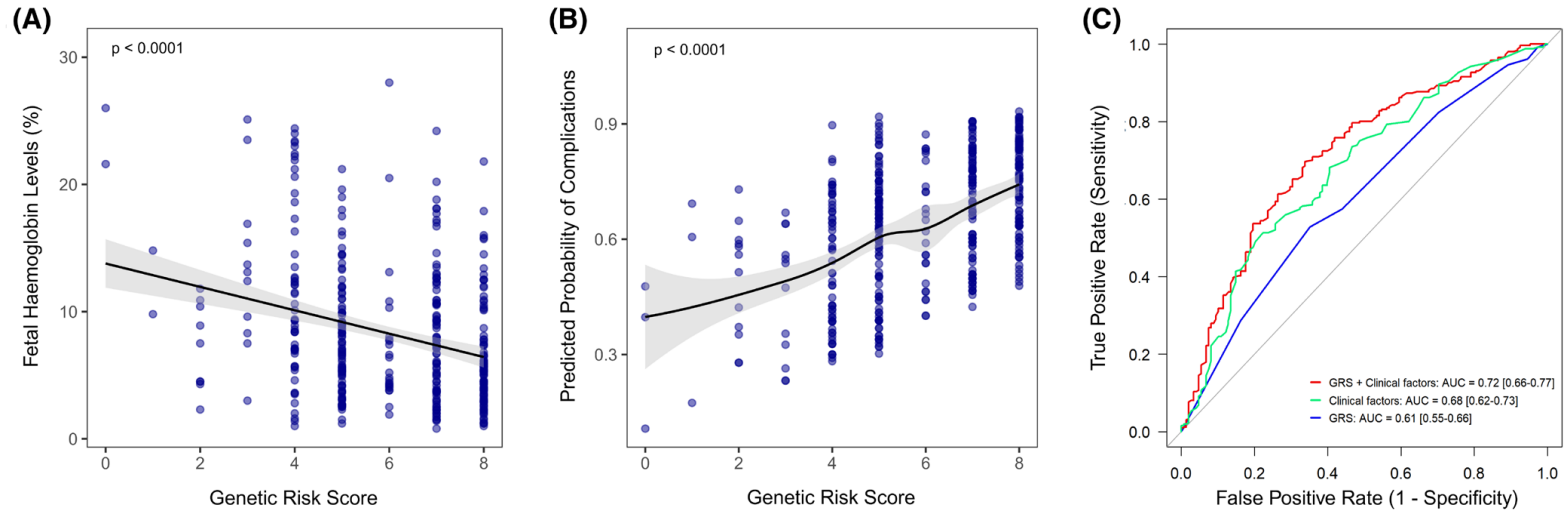
Association of the genetic risk score (GRS) with fetal haemoglobin (HbF) levels and clinical complications in sickle cell anaemia (SCA) patients. (A) Association between the GRS and HbF levels (%) in SCA patients. (B) Association between the GRS and the predicted probability of developing clinical complications in SCA patients. The black line represents the logistic regression fit with a 95% confidence interval (shaded area). Adjusted *p*‐values are presented. (C) ROC curves showing the discriminative performance for predicting clinical complications using different models. The GRS combined with clinical factors (red line) showed the highest accuracy, followed by clinical factors alone (green line) and the GRS alone (blue line).

We performed a multiple linear regression analysis to evaluate the association between HbF levels and the GRS, adjusting for age, gender and VOCs/year. The model was statistically significant (*p* < 0.0001) and explained approximately 11.5% of the variance in HbF levels. The GRS was significantly associated with HbF levels (*β* = −0.315, *p* < 0.0001), indicating that a higher GRS was associated with lower HbF levels (Figure 3A). Significant associations were also observed with RBC count, Hb levels and haematocrit (*p* < 0.0001) (Figure S4).

Moreover, we observed that a higher GRS was significantly associated with an increased likelihood of developing at least one disease complication (OR = 1.30, 95% CI: 1.15–1.47, *p* < 0.0001) (Figure 3B). After additional adjustment for baseline HbF, the GRS remained independently associated with clinical complications (OR = 1.19, 95% CI: 1.03–1.38, *p* = 0.016). The GRS was also associated with an increased risk of each clinical complication (Table S9).

The ROC curve analysis demonstrated that the model, including only the clinical factors (age, gender and VOCs/year), yielded an AUC of 0.68 (95% CI: 0.62–0.73), while the model based on the GRS presented an AUC of 0.61 (95% CI: 0.55–0.66). The multivariable model, including GRS and the clinical factors, achieved the highest discriminative performance for predicting complications, with an AUC of 0.72 (95% CI: 0.66–0.77) (Figure 3C).

Finally, based on the *BCL11A* TTA− haplotype and the *HBS1L‐MYB* G allele, the GRS regression analysis revealed that each additional point in the GRS was associated with a significant reduction in HbF (*β* = −0.347; *p* < 0.0001), accounting for 13.7% of the variance in HbF. The GRS was associated with the overall risk of developing at least one of the five evaluated complications (OR = 1.76, 95% CI 1.32–2.35, *p* < 0.0001). Moreover, higher GRS values were significantly associated with an increased risk of stroke (OR = 1.86, *p* = 0.007), AVN (OR = 1.66, *p* = 0.018), LUs (OR = 1.87, *p* = 0.003) and ACS (OR = 1.56, *p* = 0.033) (Table S8).

## DISCUSSION

The genetic modulation of HbF levels in SCA has been widely studied, with *BCL11A* and *HBS1L‐MYB* polymorphisms among the most strongly associated determinants.^10^, ^11^, ^24^, ^25^, ^26^, ^27^, ^28^, ^29^ In this study, we integrated these HbF‐modifying loci into a GRS and evaluated their combined impact on disease severity in a well‐characterized Brazilian SCA cohort.

Genetic variants influencing HbF levels in the haematopoietic transcription factor *BCL11A* were first identified through genome‐wide association studies (GWAS),^30^, ^31^ with several functional SNPs located within the transcriptionally active intron 2 region.^7^ In addition, GWAS have identified polymorphisms in the *HBS1L‐MYB* intergenic region as major modifiers of HbF levels, particularly variants within the HMIP‐2 block, which show a robust association with HbF levels.^15^, ^18^, ^32^ Here, individuals carrying variant alleles exhibited higher HbF levels, which may contribute to a milder clinical course of SCA. Specifically, the *HBS1L‐MYB* rs9399137 variant demonstrated the most substantial effect, accounting for 14.9% of the variance in HbF levels, which is higher than the reported elsewhere.^9^, ^11^, ^27^, ^33^, ^34^ Notably, in prior observations, the variants with the highest contribution to HbF variance were the same as those analysed here, reinforcing their significant roles.^9^, ^10^, ^11^, ^35^

The strong association between *BCL11A* and *HBS1L‐MYB* variants with HbF levels aligns with previous studies while also highlighting some differences. In our analysis, rs4671393 explained 6.5% of the HbF variance, compared to previously reported values ranging from 10% to 14.1% in African American and Brazilian cohorts.^10^, ^11^, ^35^ rs11886868 accounted for the lowest variance in our study (5.45%), whereas earlier studies reported a range from 4.5% to 12%.^9^, ^10^, ^11^ These discrepancies suggest that genetic admixture has a significant influence on the genetic architecture of HbF regulation across populations. This is particularly relevant in the Brazilian context, where extensive admixture likely contributes to the variability in the proportion of HbF variance explained by these SNPs. The Brazilian population background is characterized primarily by African and European populations, which may influence both the distribution and effect size of HbF‐modifying variants (Table S10). Most large studies to date have focused on African, African American or European‐derived cohorts, whereas data from highly admixed populations remain limited. In this context, our findings provide valuable evidence that *BCL11A* and *HBS1L‐MYB* variants exert robust effects on HbF levels and clinical severity in a Brazilian cohort, supporting the relevance of these loci beyond previously studied populations. Additionally, we provide data from an SCA population in which the *XmnI* polymorphism is extremely rare, and the CAR haplotype is predominant.

Previous evidence showed that HbF‐boosting variants influence the occurrence of clinical complications in SCA.^10^, ^11^, ^24^, ^25^, ^26^ Lettre et al. (2008) reported an association with VOC episodes.^10^ In contrast, homozygosity for ancestral alleles of rs9399137 and rs4671393 was linked to increased ACS risk and infection‐related hospitalizations.^11^ Notably, carriers of both the *BCL11A* and HMIP‐2 HbF‐boosting haplotypes exhibited higher HbF levels and fewer complications in paediatric SCA patients.^36^ Here, we demonstrated that genetic variants in *BCL11A* and *HBS1L‐MYB* are associated with the risk and age at onset of adverse clinical outcomes in SCA. Individuals with low‐HbF genotypes showed a higher risk of severe complications, including stroke, AVN, priapism, ACS and LUs, indicating that the lack of HbF‐boosting alleles contributes to lower HbF levels and a more severe clinical course. These findings reinforce the impact of HbF as a major modifier of SCA phenotype and highlight the polygenic nature of HbF regulation and its downstream impact on clinical manifestations.

Consistent with this, the *BCL11A* haplotype analysis showed that each additional copy of the haplotype carrying the HbF‐boosting alleles (TCA) was associated with an increase in HbF levels, while the non‐HbF‐boosting haplotype (TCA−) was associated with an increased risk of multiple clinical complications. Importantly, although the three *BCL11A* SNPs reside within the same LD block, the combination of high *D*′ and LOD scores with variable *r* ^2^ values indicates a shared ancestral origin without complete redundancy, supporting the relevance of analysing their individual and combined effects on HbF levels.

To evaluate the cumulative impact of these risk alleles, we constructed a GRS with the four variants analysed in this study. The GRS was strongly associated with HbF levels and the overall risk of SCA complications. An increasing number of risk alleles contribute to lower HbF levels and a higher probability of experiencing disease‐related complications, emphasizing the cumulative effect of HbF‐related genetic modifiers on SCA severity. Several studies have demonstrated that polygenic scores, based on the cumulative number of HbF‐boosting alleles, are associated with increased HbF levels as well as increased haemoglobin, reduced haemolysis and fewer transfusions.^9^, ^33^, ^37^, ^38^, ^39^ In addition, these scores have been linked to key clinical outcomes in SCA, including fewer hospitalizations, lower rates of stroke and ACS, reduced frequency of VOCs and an overall decrease in the number of disease‐related complications.^9^, ^33^, ^38^, ^39^, ^40^

Our findings are consistent with previous studies and extend them by demonstrating a clear association between the GRS and multiple clinical complications of SCA. ROC curve analyses further showed that integrating genetic information with conventional clinical factors improves the prediction of complication risk. Although the GRS was not validated in an independent cohort, these results suggest that incorporating HbF‐related genetic scores alongside clinical risk factors may improve the early identification of high‐risk patients. In a broader context, understanding genetic modifiers may also have therapeutic implications, as illustrated by emerging gene therapy approaches targeting erythroid *BCL11A* expression.^41^ While such therapies are not yet widely accessible, knowledge of HbF‐modifying loci may contribute to improved risk stratification and targeted management strategies.

Whereas HbF levels remain the most informative and accessible biomarker of disease severity in SCA, genetic summary measures provide complementary insight, particularly in clinical situations where baseline HbF levels are unavailable or difficult to interpret, such as early childhood before stabilization of basal HbF levels, early initiation of HU therapy or in patients undergoing chronic transfusion therapy. In addition, the HbF‐based GRS helps explain individual variability in HbF levels across populations and may provide a useful tool for understanding heterogeneity in clinical response to HU therapy.^7^

Even though these genetic variants influence HbF levels, a significant proportion of HbF variability remains unexplained by current models. This missing heritability may be attributed to other gene–gene interactions, epigenetic modifications or the presence of multiple rare variants with small effect sizes.^37^ However, despite these limitations, approaches that evaluate the combined effect of multiple variants, along with other modifying factors, provide a more comprehensive and informative assessment of genetic contributions to disease severity compared to single‐SNP analyses.

In summary, our results provide valuable insights into the role of well‐established *BCL11A* and *HBS1L‐MYB* polymorphisms in affecting SCA severity, reinforcing their clinical significance beyond HbF regulation. By demonstrating strong associations between these variants and major SCA complications in a well‐characterized cohort, our study highlights their combined contribution to key causes of morbidity and mortality in SCA. Additional studies integrating genetic, environmental and therapeutic factors are essential to deepen our understanding of SCA heterogeneity and the genetic regulation of HbF.

## AUTHOR CONTRIBUTIONS

G.S.A. performed experiments, statistical analyses, interpreted data and drafted the manuscript. A.P.S., M.V.D., I.F.D., D.A.P.‐M., A.B.A. and T.S.S.F. updated the clinical data, interpreted data and reviewed the manuscript. E.B.‐J. performed statistical analyses and reviewed the manuscript. A.C.A. and A.S.A. recruited patients, assured access to patients' samples and updated the clinical data. S.T.O.S., F.F.C., A.R.L.‐A. and M.A.C.B. interpreted data and reviewed the manuscript. M.A.C.B. conceived and designed the study and gave the final approval of the version to be submitted.

## FUNDING INFORMATION

This work was supported by Conselho Nacional de Desenvolvimento Científico e Tecnológico (CNPq, Grant #405918/2022‐4), (CNPq, Grant #408710/2021‐7), and Fundação de Amparo à Ciência e Tecnologia de Pernambuco (FACEPE Grant #APQ‐1212‐2.02/22).

## CONFLICT OF INTEREST STATEMENT

The authors have no competing financial interests to declare.

## Supporting information


Data S1.

## Data Availability

The data that support the findings of this study are available from the corresponding author upon reasonable request.

## References

[1] Piccin A , Murphy C , Eakins E , Rondinelli M , Daves M , Vecchiato C , et al. Insight into the complex pathophysiology of sickle cell anemia and possible treatment. Eur J Haematol. 2019;102:319–330. 10.1111/ejh.13212 30664257

[2] Akinsheye I , Alsultan A , Solovieff N , Ngo D , Baldwin CT , Sebastiani P , et al. Fetal hemoglobin in sickle cell anemia. Blood. 2011;118:19–27. 10.1182/blood-2011-03-325258 21490337 PMC3139383

[3] Bhatnagar P , Keefer J , Casella J , Barron‐Casella E , Bean C , Hooper C , et al. Association between baseline fetal hemoglobin levels and incidence of severe vaso‐occlusive pain episodes in children with sickle cell anemia. Pediatr Blood Cancer. 2013;60:125–127. 10.1002/pbc PMC4387562 23677903

[4] Platt OS . Hydroxyurea for the treatment of sickle cell anemia. N Engl J Med. 2008;358:1362–1369. 18367739 10.1056/NEJMct0708272

[5] Sankaran VG , Orkin SH . The switch from fetal to adult hemoglobin. Cold Spring Harb Perspect Med. 2013;3:a011643. 10.1101/cshperspect.a011643 23209159 PMC3530042

[6] Liu N , Hargreaves VV , Zhu Q , Kurland JV , Hong J , Kim W , et al. Direct promoter repression by BCL11A controls the fetal to adult hemoglobin switch. Cell. 2018;173:430–442.e17. 10.1016/j.cell.2018.03.016 29606353 PMC5889339

[7] Menzel S , Thein SL . Genetic modifiers of fetal haemoglobin in sickle cell disease. Mol Diagn Ther. 2019;23:235–244. 10.1007/s40291-018-0370-8 30478714

[8] Mohammad SNNA , Iberahim S , Wan Ab Rahman WS , Hassan MN , Edinur HA , Azlan M , et al. Single nucleotide polymorphisms in *XMN1‐HBG2*, *HBS1L‐MYB*, and *BCL11A* and their relation to high fetal hemoglobin levels that alleviate anemia. Diagnostics. 2022;12:1–13. 10.3390/diagnostics12061374 PMC9221560 35741184

[9] Leonardo FC , Brugnerotto AF , Domingos IF , Fertrin KY , de Albuquerque DM , Bezerra MAC , et al. Reduced rate of sickle‐related complications in Brazilian patients carrying HbF‐promoting alleles at the *BCL11A* and *HMIP‐2* loci. Br J Haematol. 2016;173:456–460. 10.1111/bjh.13961 26888013

[10] Lettre G , Sankaran VG , Bezerra MAC , Araujo AS , Uda M , Sanna S , et al. DNA polymorphisms at the *BCL11A*, *HBS1L‐MYB*, and β‐globin loci associate with fetal hemoglobin levels and pain crises in sickle cell disease. Proc Natl Acad Sci USA. 2008;105:11869–11874. 10.1073/pnas.0804799105 18667698 PMC2491485

[11] Sales RR , Belisário AR , Faria G , Mendes F , Luizon MR , Viana MB . Functional polymorphisms of *BCL11A* and *HBS1L‐MYB* genes affect both fetal hemoglobin level and clinical outcomes in a cohort of children with sickle cell anemia. Ann Hematol. 2020;99:1453–1463. 10.1007/s00277-020-04079-2 32447424

[12] Xu J , Bauer DE , Kerenyi MA , Vo TD , Hou S , Hsu YJ , et al. Corepressor‐dependent silencing of fetal hemoglobin expression by BCL11A. Proc Natl Acad Sci USA. 2013;110:6518–6523. 10.1073/pnas.1303976110 23576758 PMC3631619

[13] Bauer DE , Kamran SC , Lessard S , Xu J , Fujiwara Y , Lin C , et al. An erythroid enhancer of *BCL11A* subject to genetic variation determines fetal hemoglobin level. Science (1979). 2013;342:253–257. 10.1126/science.1242088.An PMC4018826 24115442

[14] Sankaran VG , Menne TF , Xu J , Akie TE , Lettre G , Van Handel B , et al. Human fetal hemoglobin expression is regulated by the developmental stage‐specific repressor *BCL11A* . Science. 2008;322:1839–1842. 19056937 10.1126/science.1165409

[15] Sales RR , Nogueira BL , Tosatti JAG , Gomes KB , Luizon MR . Do genetic polymorphisms affect fetal hemoglobin (HbF) levels in patients with sickle cell anemia treated with hydroxyurea? A systematic review and pathway analysis. Front Pharmacol. 2022;12:1–13. 10.3389/fphar.2021.779497 PMC8814522 35126118

[16] Bianchi E , Zini R , Salati S , Tenedini E , Norfo R , Tagliafico E , et al. c‐myb supports erythropoiesis through the transactivation of *KLF1* and *LMO2* expression. Blood. 2010;116:99–111. 10.1182/blood-2009-08-238311 20686118

[17] Wang X , Angelis N , Thein SL . MYB—a regulatory factor in hematopoiesis. Gene. 2018;665:6–17. 10.1016/j.gene.2018.04.065 29704633 PMC10764194

[18] Stadhouders R , Aktuna S , Thongjuea S , Aghajanirefah A , Pourfarzad F , Van IJcken W , et al. *HBS1L‐MYB* intergenic variants modulate fetal hemoglobin via long‐range MYB enhancers. J Clin Invest. 2014;124:1699–1710. 10.1172/JCI71520 24614105 PMC3973089

[19] Davis L , Dibner M , Battey J . Basic methods in molecular biology (1st ed.). New York: Elsevier; 1986. 10.1016/0968-0004(87)90140-x

[20] Powars DR . Beta s‐gene‐cluster haplotypes in sickle cell anemia. Clinical and hematologic features. Hematol Oncol Clin North Am. 1991;5:475–493. 1713910

[21] Dodé C , Krishnamoorthy R , Lamb J , Rochette J . Rapid analysis of ‐alpha 3.7 thalassaemia and alpha alpha alpha anti 3.7 triplication by enzymatic amplification analysis. Br J Haematol. 1992;82:105–111. 10.1111/j.1365-2141.1993.tb04639.x 8435317

[22] Barrett JC , Fry B , Maller J , Daly MJ . Haploview: analysis and visualization of LD and haplotype maps. Bioinformatics. 2005;21(2):263–265. 10.1093/bioinformatics/bth457 15297300

[23] Anderson CA , Pettersson FH , Clarke GM , Cardon LR , Morris AP , Zondervan KT . Data quality control in genetic case‐control association studies. Nat Protoc. 2010;5(9):1564–1573. 10.1038/nprot.2010.116 21085122 PMC3025522

[24] Upadhye D , Jain D , Trivedi Y , Nadkarni A , Ghosh K , Colah R . Influence of single nucleotide polymorphisms in the *BCL11A* and *HBS1L‐MYB* gene on the HbF levels and clinical severity of sickle cell anaemia patients. Ann Hematol. 2016;95:1201–1203. 10.1007/s00277-016-2675-1 27098811

[25] Chaouch L , Moumni I , Ouragini H , Darragi I , Kalai M , Chaouachi D , et al. rs11886868 and rs4671393 of *BCL11A* associated with HbF level variation and modulate clinical events among sickle cell anemia patients. Hematology (United Kingdom). 2016;21:425–429. 10.1080/10245332.2015.1107275 27077760

[26] El‐Ghamrawy M , Yassa ME , Tousson AMS , El‐hady MA , Mikhaeil E , Mohamed NB , et al. Association between *BCL11A*, *HSB1L‐MYB*, and *XmnI* γG‐158 (C/T) gene polymorphism and hemoglobin F level in Egyptian sickle cell disease patients. Ann Hematol. 2020;99:2279–2288. 10.1007/s00277-020-04187-z 32772141

[27] Brahim AT , Taleb M , Soumaré H , Ghaber SM , Mohamed A , Boukhary AOMS . Genotyping the *BCL11A* single nucleotide polymorphism and associated levels of fetal hemoglobin in Mauritanian sickle cell patients. Front Biosci Sch. 2024;16:11. 10.31083/j.fbs1602011 38939975

[28] Akbulut‐Jeradi N , Fernandez MJ , Al Khaldi R , Sukumaran J , Adekile A . Unique polymorphisms at *BCL11A*, *HBS1L‐MYB* and *HBB loci* associated with HbF in Kuwaiti patients with sickle cell disease. J Pers Med. 2021;11:1–11. 10.3390/jpm11060567 PMC8234980 34204365

[29] Adeyemo TA , Ojewunmi OO , Oyetunji IA , Rooks H , Rees DC , Akinsulie AO , et al. A survey of genetic fetal‐haemoglobin modifiers in Nigerian patients with sickle cell anaemia. PLoS One. 2018;13:1–10. 10.1371/journal.pone.0197927 PMC5991720 29879141

[30] Menzel S , Garner C , Gut I , Matsuda F , Yamaguchi M , Heath S , et al. A QTL influencing F cell production maps to a gene encoding a zinc‐finger protein on chromosome 2p15. Nat Genet. 2007;39:1197–1199. 10.1038/ng2108 17767159

[31] Uda M , Galanello R , Sanna S , Lettre G , Sankaran VG , Chen W , et al. Genome‐wide association study shows *BCL11A* associated with persistent fetal hemoglobin and amelioration of the phenotype of beta‐thalassemia. Proc Natl Acad Sci. 2008;105:1620–1625. 10.1073/pnas.0711566105 18245381 PMC2234194

[32] Thein SL , Menzel S , Peng X , Best S , Jiang J , Close J , et al. Intergenic variants of *HBS1L‐MYB* are responsible for a major quantitative trait locus on chromosome 6q23 influencing fetal hemoglobin levels in adults. Proc Natl Acad Sci USA. 2007;104:11346–11351. 10.1073/pnas.0611393104 17592125 PMC2040901

[33] Al‐Allawi N , Qadir SMA , Puehringer H , Chui DHK , Farrell JJ , Oberkanins C . The association of *HBG2*, *BCL11A*, and *HMIP* polymorphisms with fetal hemoglobin and clinical phenotype in Iraqi Kurds with sickle cell disease. Int J Lab Hematol. 2019;41:87–93. 10.1111/ijlh.12927 30216683

[34] Wonkam A , Ngo Bitoungui VJ , Vorster AA , Ramesar R , Cooper RS , Tayo B , et al. Association of variants at *BCL11A* and *HBS1L‐MYB* with hemoglobin F and hospitalization rates among sickle cell patients in Cameroon. PLoS One. 2014;9:e92506. 10.1371/journal.pone.0092506 24667352 PMC3965431

[35] Cardoso GL , Diniz IG , Martins da Silva ANL , Cunha DA , da Silva Junior JS , Carvalho UchÔa CT , et al. DNA polymorphisms at *BCL11A*, *HBS1L‐MYB* and *Xmn1‐HBG2* site loci associated with fetal hemoglobin levels in sickle cell anemia patients from northern Brazil. Blood Cells Mol Dis. 2014;53:176–179. 10.1016/j.bcmd.2014.07.006 25084696

[36] Sales RR , Nogueira BL , Belisário AR , Faria G , Mendes F , Viana MB , et al. Fetal hemoglobin‐boosting haplotypes of *BCL11A* gene and *HBS1L‐MYB* intergenic region in the prediction of clinical and hematological outcomes in a cohort of children with sickle cell anemia. J Hum Genet. 2022;67:701–709. 10.1038/s10038-022-01079-0 36167770

[37] Milton JN , Gordeuk VR , Taylor JG , Gladwin MT , Steinberg MH , Sebastiani P . Prediction of fetal hemoglobin in sickle cell anemia using an ensemble of genetic risk prediction models. Circ Cardiovasc Genet. 2014;7:110–115. 10.1161/CIRCGENETICS.113.000387 24585758 PMC3994553

[38] Gardner K , Fulford T , Silver N , Rooks H , Angelis N , Allman M , et al. G(HbF): a genetic model of fetal hemoglobin in sickle cell disease. Blood Adv. 2018;2:235–239. 10.1182/bloodadvances.2017009811 29437638 PMC5812320

[39] Pincez T , Lo KS , d'Alexandry d'Orengiani ALPH , Garrett ME , Brugnara C , Ashley‐Koch AE , et al. Variation and impact of polygenic hematologic traits in monogenic sickle cell disease. Haematologica. 2023;108:870–881. 10.3324/haematol.2022.281180 36226494 PMC9973495

[40] Rampersaud E , Kang G , Palmer LE , Rashkin SR , Wang S , Bi W , et al. A polygenic score for acute vaso‐occlusive pain in pediatric sickle cell disease. Blood Adv. 2021;5:2839–2851. 10.1182/bloodadvances.2021004634 34283174 PMC8341359

[41] Kirkham JK , Estepp JH , Weiss MJ , Rashkin SR . Genetic variation and sickle cell disease severity: a systematic review and meta‐analysis. JAMA Netw Open. 2023;6(10):e2337484. 10.1001/jamanetworkopen.2023.37484 37851445 PMC10585422

