# Supplementary material for: A genetic risk score based on BCL11A and HBS1L‐MYB variants predicts clinical severity in Brazilian sickle cell anaemia patients
Source: Br J Haematol. 2026 Apr 16;208(6):2203–11. doi: 10.1111/bjh.70489 (PMC13267477; doi:10.1111/bjh.70489)
Supplement: Supplementary file 1 — Data S1. [file BJH-208-2203-s001.docx]

**SUPPLEMENTAL DATA**

**A genetic risk score based on *BCL11A* and *HBS1L-MYB* variants predicts clinical severity in Brazilian sickle cell anaemia patients**

Gabriela S Arcanjo^1,2^, Alexsandro P Silva^1^, Madi V Diniz^1^, Igor F Domingos^3^, Diego A Pereira-Martins^4^, Amanda B Araújo^1^, Talita S S França^1^; Ana C Anjos^1,5^, Aderson S Araujo^5^, Edis Belini-Junior^6^, Sara T O Saad^2^, Fernando F Costa^2^, Antonio R Lucena-Araujo^1^, Marcos André C Bezerra^1^

**Affiliations:**

^1^ Genetics and Molecular Biology Postgraduate Program, Federal University of Pernambuco, Recife, Pernambuco, Brazil;

^2^ Hematology and Hemotherapy Center, State University of Campinas, Campinas, São Paulo, Brazil

^3^ Cardiology Emergency Unit of Pernambuco, University of Pernambuco, Recife, Pernambuco, Brazil;

^4^ Department of Hematology, Cancer Research Centre Groningen, University Medical Centre Groningen, University of Groningen, Groningen, the Netherlands;

^5^ Department of Internal Medicine, Hematology and Hemotherapy Foundation of Pernambuco, Recife, Pernambuco, Brazil;

^6^ Federal University of Mato Grosso do Sul (UFMS), Molecular Biology and Genetics Laboratory, Três Lagoas, MS, Brazil

* Corresponding Author: Marcos A C Bezerra. Email: [macbezerra.ufpe@gmail.com](mailto:macbezerra.ufpe@gmail.com)

**SUPPLEMENTAL METHODS**

*Clinical complications definition*

Clinical complications were identified through review of medical records. All patients were followed at a single reference centre and managed by haematology clinicians. Clinical diagnoses and management followed established national and international guidelines. The occurrence of clinical complications was assessed over the entire period of available clinical follow-up for each patient, defined as the time from admission to the haematology centre until the most recent medical record review in 2023. The median follow-up duration was 24 years (range: 10–45 years).

The definitions of the complications were as follows: Stroke was defined as a focal neurological deficit resulting from regional cerebral blood flow impairment due to stenosis or occlusion of cerebral vasculature, confirmed by neuroimaging (computed tomography or magnetic resonance imaging) (1). It should be noted that silent cerebral infarcts could not be systematically excluded, as routine brain magnetic resonance imaging is not performed in the Brazilian public healthcare system due to cost constraints. Acute chest syndrome was diagnosed by the presence of a new pulmonary infiltrate on chest radiography involving at least one lung segment, in combination with one or more of the following clinical features: fever, chest pain, tachypnoea, cough, wheezing, or hypoxia (2). Avascular necrosis was defined as radiologically confirmed osteonecrosis of the femoral or humeral head. Diagnosis was established by plain radiography and/or magnetic resonance imaging, and only symptomatic cases documented in the clinical records were included (3). Leg ulcers were defined as chronic, non-healing cutaneous ulcers, typically located in the malleolar region, that failed to heal within a minimum period of six weeks (4). Only ulcers attributed to sickle cell–related vasculopathy were considered. Finally, priapism was defined as a sustained, painful penile erection lasting ≥4 hours and requiring medical evaluation (5).

*BCL11A and HBS1L-MYB genotyping*

DNA was extracted from leukocytes using the standard phenol-chloroform extraction method (6). All patients were fully genotyped for *BCL11A* rs4671393 (Assay ID: C__25926414_10), rs1427407 (Assay ID: C___8899456_10), rs11886868 (Assay ID: C__11363852_10) and *HBS1L-MYB* intergenic region rs9399137 (Assay ID: C__30488126_20) using real-time PCR with TaqMan® probes (Applied Biosystems, Foster City, CA, USA). Each reaction had a final volume of 5 µL, containing 2.5 μL of TaqMan Genotyping Master Mix (Applied Biosystems, Foster City, USA), 0.25 μL of 20× TaqMan probes, 1.25 μL of ultrapure water, and 1 μL of DNA at 50 ng/μL. The cycling conditions consisted of 95°C for 10 minutes, followed by 40 cycles of 95°C for 15 seconds and 60°C for 1 minute in a QuantStudio™ 5 (Applied Biosystems, Foster City, USA) equipment.

*Statistical analyses*

Descriptive statistics were used to summarize baseline characteristics. Hardy–Weinberg equilibrium (HWE) for each SNP was assessed via the Chi-square test. Only variants with HWE p < 0.001 were considered for exclusion (7). All analysed SNPs showed HWE p-values above this threshold (rs4671393: p = 0.012; rs1427407: p = 0.028; rs11886868: p = 0.253; rs9399137: p = 0.142). Categorical variables were compared using Fisher’s exact or Chi-square tests, and continuous variables using Mann–Whitney or Kruskal–Wallis tests with Dunn’s post-test, when appropriate. P-values were adjusted for multiple testing using the Benjamini–Hochberg method.

Linkage disequilibrium (LD) and haplotype analyses of *BCL11A* gene SNPs were performed using Haploview software (v. 4.2). The squared correlation coefficient (r²), standardized disequilibrium coefficient D (D’), and the log of the likelihood odds ratio (LOD) score were used to calculate LD for each SNP pair. The solid spine method was applied in haplotype analyses because of high D’ values (0.89-0.95) and LOD scores (48.87-96.8), along with moderate r^2^ values (0.43-0.78) (8,9).

To assess the association between *BCL11A* and *HBS1L-MYB* gene SNPs as well as *BCL11A* haplotypes with HbF levels, we performed linear regression analyses under an additive model. Univariate and multivariate logistic regressions were used to evaluate the association between the individual SNPs and *BCLL1A* haplotypes with clinical complications. Results were expressed as odds ratios (ORs) with 95% confidence intervals (CIs). To assess the long-term risk of complications, cumulative incidence curves were constructed using the Kaplan–Meier method and the log-rank test. Multivariate Cox proportional hazards regression models were also applied to estimate hazard ratios (HRs) for time-to-event outcomes. Time was defined as the duration from cohort entry (first admission to the haematology centre) to the occurrence of the event or censoring. Multivariate regression models were constructed, adjusting for gender, age, and the number of VOCs/year.

An unweighted genetic risk score (GRS) was constructed to integrate the cumulative effect of genetic variants by summing the number of risk alleles present across the four SNPs evaluated in the study. Each SNP was assigned a score based on genotype: 2 points for homozygous risk alleles, 1 point for heterozygous, and 0 points for homozygous non-risk alleles (HbF-boosting alleles). The association between GRS, haematological parameters, and the risk of complications was assessed using regression models adjusted for age, gender, and VOCs/year. For comparison, a GRS was also constructed by assigning one point for each copy of high-risk *BCL11A* haplotypes and the *HBS1L-MYB* rs9399137 risk allele, resulting in a cumulative score ranging from 0 to 4, which was subsequently evaluated for associations with HbF levels and clinical complications in regression models.

To assess the discriminatory capacity of the GRS for predicting complications, we conducted ROC curve analyses. Three logistic regression models were constructed: a univariable model with only the GRS, a clinical model including age, gender, and VOCs/year, and a multivariable model combining the GRS with clinical variables. Predicted probabilities from each model were used to generate ROC curves with the *pROC* package in R. Model performance was evaluated using the AUC and corresponding 95% CI.

All statistical analyses were performed using SPSS Statistics 19.0 (IBM Corporation, Somers, NY, USA) and the R software (The CRAN project, www.r-project.org). A significance level of 5% (p < 0.05) was considered statistically significant.

**References:**

1. Ohene-frempong K, Weiner SJ, Sleeper LA, Miller ST, Embury S, Moohr JW, et al. Cerebrovascular Accidents in Sickle Cell Disease: Rates and Risk Factors. Blood. 1998;91(1):288–95.

2. Desai P, Ataga K. The acute chest syndrome of sickle cell disease. Expert Opin Pharmacother. 2013;14(8):991–9.

3. Adesina OO, Neumayr LD. Osteonecrosis in sickle cell disease: an update on risk factors, diagnosis, and management. Hematology. 2018;351–8.

4. Minniti CP, Eckman J, Sebastiani P, Steinberg MH, Ballas SK. Leg ulcers in sickle cell disease. Am J Hematol. 2010;85(10):831–3.

5. Idris IM, Burnett AL, Debaun MR. Epidemiology and treatment of priapism in sickle cell disease. Hematology. 2022;450–2.

6. Davis L, Dibner M, Battey J. Basic Methods in Molecular Biology. Vol. 1rst ed., Elsevier. 1986. 1–399 p.

7. Anderson CA, Pettersson FH, Clarke GM, Cardon LR, Morris AP, Zondervan KT. Data quality control in genetic case-control association studies. Nat Protoc. 2010;5(9):1564–73.

8. Gabriel SB, Schaffner SF, Nguyen H, Moore JM, Roy J, Blumenstiel B, et al. The structure of haplotype blocks in the human genome. Science (80- ). 2002;296(5576):2225–9.

9. Barrett JC, Fry B, Maller J, Daly MJ. Haploview: Analysis and visualization of LD and haplotype maps. Bioinformatics. 2005;21(2):263–5.

**Supplementary Table S1.** Association between baseline fetal haemoglobin (HbF) levels and clinical complications in patients with sickle cell anaemia.

|  | **Fetal Haemoglobin Levels** | | |
| --- | --- | --- | --- |
| **Clinical Complications** | **Yes** | **No** | **p-value** |
| Stroke | 4.4 (3.6) | 9.3 (9.2) | <0.0001 |
| Avascular necrosis | 6.5 (6.4) | 9.3 (9.2) | 0.001 |
| Leg ulcers | 6.0 (5.5) | 9.3 (9.2) | <0.0001 |
| Priapism* | 6.6 (5.3) | 9.4 (10.0) | 0.042 |
| Acute chest syndrome | 6.7 (6.5) | 9.3 (9.2) | 0.002 |
| *Includes only male individuals.  Fetal Haemoglobin is described as median (interquartile range (IQR)). | | | |

**Supplementary Table S2**. Linkage disequilibrium analysis of the *BCL11A* locus.

| **SNPs** | **r^2^** | **D’** | **D’ confidence bounds** | **LOD** |
| --- | --- | --- | --- | --- |
| rs1427407 - rs11886868 | 0.438 | 0.89 | 0.82-0.94 | 48.8 |
| rs1427407 - rs4671393 | 0.789 | 0.91 | 0.87-0.95 | 96.8 |
| rs11886868 - rs4671393 | 0.527 | 0.94 | 0.89-0.98 | 63.3 |
| r^2^, squared correlation coefficient; D’, standardized disequilibrium coefficient D; LOD, log of the likelihood odds ratio. | | | | |

| **Supplementary table S3.** Univariate and multivariate binary logistic regression for stroke development based on *BCL11A* and *HBS1L-MYB* variants. | | | | | | | | |
| --- | --- | --- | --- | --- | --- | --- | --- | --- |
| **Gene**  **SNP** | **Comparison** | **Genotypes** | **Stroke**  **N (%)** | **Non-stroke**  **N (%)** | **Unadjusted**  **OR 95% CI** | **Unadjusted**  ***p-value*** | **Adjusted**  **OR 95% CI** | **Adjusted**  ***p-value*** |
| *BCL11A* rs4671393 | GG vs GA/AA | GG | 35 (58.3) | 62 (41.9) | 1.00 |  |  |  |
|  |  | GA/AA | 25 (41.7) | 86 (58.1) | 1.94 (1.0-3.56) | 0.032* | 2.11 (1.13-3.93) | 0.019* |
|  | AA vs GA/GG | GA/GG | 57 (95.0) | 139 (93.9) | 1.00 |  |  |  |
|  |  | AA | 03 (5.0) | 09 (6.1) | 0.81 (0.21-3.11) | 0.762 | 0.78 (0.20-3.04) | 0.724 |
|  | GG vs GA vs AA | GG | 35 (58.3) | 62 (41.9) |  |  |  |  |
|  |  | GA | 22 (36.7) | 77 (52.0) | 0.59 (0.35-1.01) | 0.057 | 0.56 (0.32-0.97) | 0.040* |
|  |  | AA | 03 (5.0) | 09 (6.1) |  |  |  |  |
| *BCL11A* rs1427407 | GG vs GT/TT | GG | 34 (56.7) | 63 (42.6) | 1.00 |  |  |  |
|  |  | GT/TT | 26 (43.3) | 85 (57.4) | 1.76 (0.96-3.23) | 0.066 | 1.93 (1.03-3.59) | 0.037* |
|  | TT vs GG/GT | GG/GT | 57 (95.0) | 141 (95.3) | 1.00 |  |  |  |
|  |  | TT | 03 (5.0) | 07 (4.7) | 1.06 (0.26-4.24) | 0.934 | 0.90 (0.21-3.74) | 0.886 |
|  | GG vs GT vs TT | GG | 34 (56.7) | 63 (42.6) |  |  |  |  |
|  |  | GT | 23 (38.3) | 78 (52.7) | 0.65 (0.38-1.12) | 0.123 | 0.60 (0.34-1.05) | 0.076* |
|  |  | TT | 03 (5.0) | 07 (4.7) |  |  |  |  |
| *BCL11A*  rs11886868 | TT vs TC/CC | TT | 22 (36.7) | 44 (29.7) | 1.00 |  |  |  |
|  |  | TC/CC | 38 (63.3) | 104 (70.3) | 1.36 (0.72-2.57) | 0.331 | 1.44 (0.75-2.75) | 0.263 |
|  | CC vs TC/TT | TT/TC | 53 (88.3) | 122 (82.4) | 1.00 |  |  |  |
|  |  | CC | 07 (11.7) | 26 (17.6) | 0.62 (0.25-1.51) | 0.295 | 0.56 (0.22-1.40) | 0.220 |
|  | TT vs TC vs CC | TT | 22 (36.7) | 44 (29.7) |  |  |  |  |
|  |  | TC | 31 (51.7) | 78 (52.7) | 0.74 (0.47-1.80) | 0.213 | 0.73 (0.45-1.17) | 0.197 |
|  |  | CC | 07 (11.7) | 26 (17.6) |  |  |  |  |
| *HBS1L-MYB* rs9399137 | TT vs TC/CC | TT | 52 (86.7) | 95 (64.2) | 1.00 |  |  |  |
|  |  | TC/CC | 08 (13.3) | 53 (35.8) | 3.62 (1.60-8.20) | 0.002* | 3.63 (1.58-8.32) | 0.002* |
|  | CC vs TC/TT | TT/TC | 60 (100) | 141 (95.3) | 1.00 |  |  |  |
|  |  | CC | 0 (0) | 07 (4.7) | 2.25 (1.04-4.57) | 0.03* | -- | -- |
|  | TT vs TC vs CC | TT | 52 (86.7) | 95 (64.2) |  |  |  |  |
|  |  | TC | 08 (13.3) | 46 (31.1) | 0.28 (0.13-0.62) | 0.002* | 0.29 (0.13-0.63) | 0.002* |
|  |  | CC | 0 (0) | 07 (4.7) |  |  |  |  |
| Adjustments in the multivariate model include age, gender, and vaso-occlusive crises (VOCs) per year as covariates. Results are reported as odds ratios (OR) with 95% confidence intervals (CI) and corresponding p-values, where OR >1 indicates increased risk, while OR <1 suggests a protective effect for the first category listed. For the additive model OR represents the effect per additional copy of the minor allele. Statistically significant associations (p < 0.05) are highlighted. | | | | | | | | |

**Supplementary table S4.** Univariate and multivariate binary logistic regression for avascular necrosis (AVN) development based on *BCL11A* and *HBS1L-MYB* genetic variants.

| **Gene**  **SNP** | **Comparison** | **Genotypes** | **AVN**  **N (%)** | **Non-AVN**  **N (%)** | **Unadjusted**  **OR 95% CI** | **Unadjusted**  ***p-value*** | **Adjusted**  **OR 95% CI** | **Adjusted**  ***p-value*** |
| --- | --- | --- | --- | --- | --- | --- | --- | --- |
| *BCL11A* rs4671393 | GG vs GA/AA | GG | 36 (54.5) | 62 (41.9) | 1.00 |  |  |  |
|  |  | GA/AA | 30 (45.5) | 86 (58.1) | 1.66 (0.92-2.98) | 0.087 | 2.05 (1.08-3.89) | 0.027* |
|  | AA vs GA/GG | GA/GG | 61 (92.4) | 139 (93.9) | 1.00 |  |  |  |
|  |  | AA | 05 (7.6) | 09 (6.1) | 1.26 (0.40-3.93) | 0.684 | 1.03 (0.29-3.54) | 0.963 |
|  | GG vs GA vs AA | GG | 36 (54.5) | 62 (41.9) |  |  |  |  |
|  |  | GA | 25 (37.9) | 77 (52.0) | 0.73 (0.45-1.19) | 0.216 | 0.57 (0.33-0.99) | 0.047* |
|  |  | AA | 05 (7.6) | 09 (6.1) |  |  |  |  |
| *BCL11A* rs1427407 | GG vs GT/TT | GG | 37 (56.1) | 63 (42.6) | 1.00 |  |  |  |
|  |  | GT/TT | 29 (43.9) | 85 (57.4) | 1.72 (0.95-3.09) | 0.069 | 2.16 (1.14-4.11) | 0.018* |
|  | TT vs GG/GT | GG/GT | 61 (92.4) | 141 (95.3) | 1.00 |  |  |  |
|  |  | TT | 05 (7.6) | 07 (4.7) | 1.65 (0.50-5.40) | 0.407 | 0.98 (0.26-3.64) | 0.978 |
|  | GG vs GT vs TT | GG | 37 (56.1) | 63 (42.6) |  |  |  |  |
|  |  | GT | 24 (36.4) | 78 (52.7) | 0.73 (0.44-1.21) | 0.228 | 0.54 (0.31-0.93) | 0.029* |
|  |  | TT | 05 (7.6) | 07 (4.7) |  |  |  |  |
| *BCL11A*  rs11886868 | TT vs TC/CC | TT | 23 (34.8) | 44 (29.7) | 1.00 |  |  |  |
|  |  | TC/CC | 43 (65.2) | 104 (70.3) | 1.26 (0.68-2.34) | 0.456 | 1.40 (0.72-2.74) | 0.318 |
|  | CC vs TC/TT | TT/TC | 54 (81.8) | 122 (82.4) | 1.00 |  |  |  |
|  |  | CC | 12 (18.2) | 26 (17.6) | 1.04 (0.49-2.21) | 0.914 | 0.85 (0.38-1.91) | 0.704 |
|  | TT vs TC vs CC | TT | 23 (34.8) | 44 (29.7) |  |  |  |  |
|  |  | TC | 31 (47.0) | 78 (52.7) | 0.90 (0.59-1.38) | 0.658 | 0.77 (0.49-1.22) | 0.281 |
|  |  | CC | 12 (18.2) | 26 (17.6) |  |  |  |  |
| *HBS1L-MYB* rs9399137 | TT vs TC/CC | TT | 54 (81.8) | 95 (64.2) | 1.00 |  |  |  |
|  |  | TC/CC | 12 (18.2) | 53 (35.8) | 2.51 (1.23-5.10) | 0.011* | 2.80 (1.30-6.03) | 0.008* |
|  | CC vs TC/TT | TT/TC | 64 (97.0) | 141 (95.3) | 1.00 |  |  |  |
|  |  | CC | 02 (3.0) | 07 (4.7) | 0.62 (0.12-3.11) | 0.570 | 0.96 (0.06-2.30) | 0.302 |
|  | TT vs TC vs CC | TT | 54 (81.8) | 95 (64.2) |  |  |  |  |
|  |  | TC | 10 (15.2) | 46 (31.1) | 0.48 (0.26-0.90) | 0.022* | 0.43 (0.22-0.83) | 0.012* |
|  |  | CC | 02 (3.0) | 07 (4.7) |  |  |  |  |
| Adjustments in the multivariate model include age, gender, and vaso-occlusive crises (VOCs) per year as covariates. Results are reported as odds ratios (OR) with 95% confidence intervals (CI) and corresponding p-values, where OR >1 indicates increased risk, while OR <1 suggests a protective effect for the first category listed. For the additive model OR represents the effect per additional copy of the minor allele. Statistically significant associations (p < 0.05) are highlighted. | | | | | | | | |

**Supplementary table S5.** Univariate and multivariate binary logistic regression for leg ulcer (LU) development based on *BCL11A* and *HBS1L-MYB* genetic variants.

| **Gene**  **SNP** | **Comparison** | **Genotypes** | **LU**  **N (%)** | **Non-LU**  **N (%)** | **Unadjusted**  **OR 95% CI** | **Unadjusted**  ***p-value*** | **Adjusted**  **OR 95% CI** | **Adjusted**  ***p-value*** |
| --- | --- | --- | --- | --- | --- | --- | --- | --- |
| *BCL11A* rs4671393 | GG vs GA/AA | GG | 48 (50.0) | 62 (41.9) | 1.00 |  |  |  |
|  |  | GA/AA | 48 (50.0) | 86 (58.1) | 1.66 (0.92-2.98) | 0.087 | 2.05 (1.08-3.89) | 0.027* |
|  | AA vs GA/GG | GA/GG | 90 (93.8) | 139 (93.9) | 1.00 |  |  |  |
|  |  | AA | 06 (6.3) | 09 (6.1) | 1.03 (0.35-2.99) | 0.957 | 0.90 (0.26-3.07) | 0.869 |
|  | GG vs GA vs AA | GG | 48 (50.0) | 62 (41.9) |  |  |  |  |
|  |  | GA | 42 (43.8) | 77 (52.0) | 0.80 (0.52-1.23) | 0.314 | 0.58 (0.35-0.97) | 0.040* |
|  |  | AA | 06 (6.3) | 09 (6.1) |  |  |  |  |
| *BCL11A* rs1427407 | GG vs GT/TT | GG | 52 (54.2) | 63 (42.6) | 1.00 |  |  |  |
|  |  | GT/TT | 44 (45.8) | 85 (57.4) | 1.59 (0.95-2.67) | 0.077 | 2.50 (1.37-4.58) | 0.003* |
|  | TT vs GG/GT | GG/GT | 91 (94.8) | 141 (95.3) | 1.00 |  |  |  |
|  |  | TT | 05 (5.2) | 07 (4.7) | 1.10 (0.34-3.59) | 0.866 | 0.56 (0.14-2.20) | 0.409 |
|  | GG vs GT vs TT | GG | 52 (54.2) | 63 (42.6) |  |  |  |  |
|  |  | GT | 39 (40.6) | 78 (52.7) | 0.71 (0.46-1.12) | 0.148 | 0.46 (0.27-0.78) | 0.004* |
|  |  | TT | 05 (5.2) | 07 (4.7) |  |  |  |  |
| *BCL11A*  rs11886868 | TT vs TC/CC | TT | 41 (42.7) | 44 (29.7) | 1.00 |  |  |  |
|  |  | TC/CC | 55 (57.3) | 104 (70.3) | 1.76 (1.03-3.01) | 0.038* | 2.52 (1.22-4.13) | 0.009* |
|  | CC vs TC/TT | TT/TC | 82 (85.4) | 122 (82.4) | 1.00 |  |  |  |
|  |  | CC | 14 (14.6) | 26 (17.6) | 0.80 (0.39-1.62) | 0.539 | 0.53 (0.24-1.20) | 0.131 |
|  | TT vs TC vs CC | TT | 41 (42.7) | 44 (29.7) |  |  |  |  |
|  |  | TC | 41 (42.7) | 78 (52.7) | 0.71 (0.48-1.04) | 0.080 | 0.56 (0.36-0.87) | 0.010* |
|  |  | CC | 14 (14.6) | 26 (17.6) |  |  |  |  |
| *HBS1L-MYB* rs9399137 | TT vs TC/CC | TT | 77 (80.2) | 95 (64.2) | 1.00 |  |  |  |
|  |  | TC/CC | 19 (19.8) | 53 (35.8) | 2.26 (1.23-4.13) | 0.008* | 2.42 (1.22-4.79) | 0.011* |
|  | CC vs TC/TT | TT/TC | 94 (97.9) | 141 (95.3) | 1.00 |  |  |  |
|  |  | CC | 02 (2.1) | 07 (4.7) | 0.42 (0.08-2.10) | 0.297 | 0.26 (0.04-1.59) | 0.145 |
|  | TT vs TC vs CC | TT | 77 (80.2) | 95 (64.2) |  |  |  |  |
|  |  | TC | 17 (17.7) | 46 (31.1) | 0.49 (0.29-0.84) | 0.010* | 0.45 (0.25-0.82) | 0.009* |
|  |  | CC | 02 (2.1) | 07 (4.7) |  |  |  |  |
| Adjustments in the multivariate model include age, gender, and vaso-occlusive crises (VOCs) per year as covariates. Results are reported as odds ratios (OR) with 95% confidence intervals (CI) and corresponding p-values, where OR >1 indicates increased risk, while OR <1 suggests a protective effect for the first category listed. For the additive model OR represents the effect per additional copy of the minor allele. Statistically significant associations (p < 0.05) are highlighted. | | | | | | | | |

**Supplementary table S6.** Univariate and multivariate binary logistic regression for priapism development based on *BCL11A* and *HBS1L-MYB* genetic variants.

| **Gene**  **SNP** | **Comparison** | **Genotypes** | **Priapism**  **N (%)** | **Non-priapism**  **N (%)** | **Unadjusted**  **OR 95% CI** | **Unadjusted**  ***p-value***** | **Adjusted**  **OR 95% CI** | **Adjusted**  ***p-value***** |
| --- | --- | --- | --- | --- | --- | --- | --- | --- |
| *BCL11A* rs4671393 | GG vs GA/AA | GG | 34 (54.8) | 17 (36.2) | 1.00 |  |  |  |
|  |  | GA/AA | 28 (45.2) | 30 (63.8) | 2.14 (0.98-4.66) | 0.055 | 2.29 (1.04-5.07) | 0.040* |
|  | AA vs GA/GG | GA/GG | 61 (98.4) | 46 (97.9) | 1.00 |  |  |  |
|  |  | AA | 01 (1.6) | 01 (2.1) | 0.75 (0.04-12.37) | 0.843 | 0.69 (0.04-11.5) | 0.801 |
|  | GG vs GA vs AA | GG | 34 (54.8) | 17 (36.2) |  |  |  |  |
|  |  | GA | 27 (43.5) | 29 (61.7) | 0.50 (0.24-1.04) | 0.065 | 0.48 (0.23-1.03) | 0.061 |
|  |  | AA | 01 (1.6) | 01 (2.1) |  |  |  |  |
| *BCL11A* rs1427407 | GG vs GT/TT | GG | 38 (61.3) | 16 (34.0) | 1.00 |  |  |  |
|  |  | GT/TT | 24 (38.7) | 31 (66.0) | 3.06 (1.39-6.76) | 0.005* | 3.27 (1.46-7.32) | 0.004* |
|  | TT vs GG/GT | GG/GT | 61 (98.4) | 46 (97.9) | 1.00 |  |  |  |
|  |  | TT | 01 (1.6) | 01 (2.1) | 0.75 (0.04-12.37) | 0.843 | 0.52 (0.03-9.41) | 0.664 |
|  | GG vs GT vs TT | GG | 38 (61.3) | 16 (34.0) |  |  |  |  |
|  |  | GT | 23 (37.1) | 30 (63.8) | 0.36 (0.17-0.77) | 0.009* | 0.34 (0.16-0.74) | 0.007* |
|  |  | TT | 01 (1.6) | 01 (2.1) |  |  |  |  |
| *BCL11A*  rs11886868 | TT vs TC/CC | TT | 24 (38.7) | 12 (25.5) | 1.00 |  |  |  |
|  |  | TC/CC | 38 (61.3) | 35 (74.5) | 1.84 (0.80-4.23) | 0.150 | 1.89 (0.81-4.36) | 0.136 |
|  | CC vs TC/TT | TT/TC | 54 (87.1) | 38 (80.9) | 1.00 |  |  |  |
|  |  | CC | 08 (12.9) | 09 (19.1) | 0.62 (0.22-1.76) | 0.376 | 0.56 (0.19-1.62) | 0.287 |
|  | TT vs TC vs CC | TT | 24 (38.7) | 12 (25.5) |  |  |  |  |
|  |  | TC | 30 (48.4) | 26 (55.3) | 0.65 (0.36-1.15) | 0.140 | 0.64 (0.35-1.63) | 0.145 |
|  |  | CC | 08 (12.9) | 09 (19.1) |  |  |  |  |
| *HBS1L-MYB* rs9399137 | TT vs TC/CC | TT | 45 (72.6) | 37 (78.7) | 1.00 |  |  |  |
|  |  | TC/CC | 17 (27.4) | 10 (21.3) | 0.71 (0.29-1.74) | 0.463 | 0.76 (0.30-1.90) | 0.567 |
|  | CC vs TC/TT | TT/TC | 61 (98.4) | 45 (95.7) | 1.00 |  |  |  |
|  |  | CC | 01 (1.6) | 02 (4.3) | 0.36 (0.03-4.19) | 0.421 | 0.22 (0.01-2.91) | 0.255 |
|  | TT vs TC vs CC | TT | 45 (72.6) | 37 (78.7) |  |  |  |  |
|  |  | TC | 16 (25.8) | 08 (17.0) | 1.14 (0.53-2.45) | 0.720 | 1.11 (0.50-2.45) | 0.797 |
|  |  | CC | 01 (1.6) | 02 (4.3) |  |  |  |  |
| Adjustments in the multivariate model include age, gender, and vaso-occlusive crises (VOCs) per year as covariates. Results are reported as odds ratios (OR) with 95% confidence intervals (CI) and corresponding p-values, where OR >1 indicates increased risk, while OR <1 suggests a protective effect for the first category listed. For the additive model OR represents the effect per additional copy of the minor allele. Statistically significant associations (p < 0.05) are highlighted. | | | | | | | | |

**Supplementary table S7.** Univariate and multivariate binary logistic regression for acute chest syndrome (ACS) development based on *BCL11A* and *HBS1L-MYB* genetic variants.

| **Gene**  **SNP** | **Comparison** | **Genotypes** | **ACS**  **N (%)** | **Non-ACS**  **N (%)** | **Unadjusted**  **OR 95% CI** | **Unadjusted**  ***p-value*** | **Adjusted**  **OR 95% CI** | **Adjusted**  ***p-value*** |
| --- | --- | --- | --- | --- | --- | --- | --- | --- |
| *BCL11A* rs4671393 | GG vs GA/AA | GG | 38 (55.1) | 62 (41.9) | 1.00 |  |  |  |
|  |  | GA/AA | 31 (44.9) | 86 (58.1) | 1.70 (0.95-3.02) | 0.071 | 1.94 (1.07-3.54) | 0.029* |
|  | AA vs GA/GG | GA/GG | 68 (98.6) | 139 (93.9) | 1.00 |  |  |  |
|  |  | AA | 01 (1.4) | 09 (6.1) | 0.22 (0.02-1.82) | 0.164 | 0.24 (0.03-2.02) | 0.193 |
|  | GG vs GA vs AA | GG | 38 (55.1) | 62 (41.9) |  |  |  |  |
|  |  | GA | 30 (43.5) | 77 (52.0) | 0.57 (0.34-0.96) | 0.036* | 0.47 (0.26-0.82) | 0.009* |
|  |  | AA | 01 (1.4) | 09 (6.1) |  |  |  |  |
| *BCL11A* rs1427407 | GG vs GT/TT | GG | 39 (56.5) | 63 (42.6) | 1.00 |  |  |  |
|  |  | GT/TT | 30 (43.5) | 85 (57.4) | 1.75 (0.98-3.12) | 0.056 | 2.07 (1.13-3.80) | 0.018* |
|  | TT vs GG/GT | GG/GT | 66 (95.7) | 141 (95.3) | 1.00 |  |  |  |
|  |  | TT | 03 (4.3) | 07 (4.7) | 0.91 (0.22-3.65) | 0.901 | 0.94 (0.22-3.91) | 0.939 |
|  | GG vs GT vs TT | GG | 39 (56.5) | 63 (42.6) |  |  |  |  |
|  |  | GT | 27 (39.1) | 78 (52.7) | 0.64 (0.38-1.07) | 0.092 | 0.52 (0.30-0.90) | 0.021* |
|  |  | TT | 03 (4.3) | 07 (4.7) |  |  |  |  |
| *BCL11A*  rs11886868 | TT vs TC/CC | TT | 23 (33.3) | 44 (29.7) | 1.00 |  |  |  |
|  |  | TC/CC | 46 (66.7) | 104 (70.3) | 1.18 (0.64-2.18) | 0.593 | 1.26 (0.67-2.36) | 0.463 |
|  | CC vs TC/TT | TT/TC | 67 (97.1) | 122 (82.4) | 1.00 |  |  |  |
|  |  | CC | 02 (2.9) | 26 (17.6) | 0.14 (0.03-0.60) | 0.009* | 0.12 (0.02-0.54) | 0.006* |
|  | TT vs TC vs CC | TT | 23 (33.3) | 44 (29.7) |  |  |  |  |
|  |  | TC | 44 (63.8) | 78 (52.7) | 0.63 (0.39-1.00) | 0.051 | 0.57 (0.35-0.92) | 0.023* |
|  |  | CC | 02 (2.9) | 26 (17.6) |  |  |  |  |
| *HBS1L-MYB* rs9399137 | TT vs TC/CC | TT | 53 (76.8) | 95 (64.2) | 1.00 |  |  |  |
|  |  | TC/CC | 16 (23.2) | 53 (35.8) | 1.84 (0.96-3.54) | 0.065 | 1.77 (0.90-3.49) | 0.094 |
|  | CC vs TC/TT | TT/TC | 68 (98.6) | 141 (95.3) | 1.00 |  |  |  |
|  |  | CC | 01 (1.4) | 07 (4.7) | 0.29 (0.03-2.45) | 0.260 | 0.26 (0.03-2.32) | 0.232 |
|  | TT vs TC vs CC | TT | 53 (76.8) | 95 (64.2) |  |  |  |  |
|  |  | TC | 15 (21.7) | 46 (31.1) | 0.56 (0.31-1.00) | 0.050 | 0.56 (0.31-1.03) | 0.065 |
|  |  | CC | 01 (1.4) | 07 (4.7) |  |  |  |  |
| Adjustments in the multivariate model include age, gender, and vaso-occlusive crises (VOCs) per year as covariates. Results are reported as odds ratios (OR) with 95% confidence intervals (CI) and corresponding p-values, where OR >1 indicates increased risk, while OR <1 suggests a protective effect for the first category listed. For the additive model OR represents the effect per additional copy of the minor allele. Statistically significant associations (p < 0.05) are highlighted. | | | | | | | | |

| **Clinical Complication** | ***BCL11A* TCA haplotype Risk of complication** | | ***BCL11A* TCA haplotype Cumulative risk** | | ***BCL11A* haplotype and *HBS1L-MYB* GRS Risk of complication** | |
| --- | --- | --- | --- | --- | --- | --- |
|  | **OR (95% CI)** | ***p-value*** | **HR (95% CI)** | ***p-value*** | **OR (95% CI)** | ***p-value*** |
| Stroke | 1.62 (0.92-2.84) | 0.091 | 1.50 (0.92-2.44) | 0.101 | 1.86 (1.18-2.93) | 0.007* |
| Avascular necrosis | 1.63 (0.94-2.82) | 0.077 | 1.45 (0.94-2.22) | 0.089 | 1.66 (1.09-2.53) | 0.018* |
| Leg Ulcers | 2.01 (1.19-3.40) | 0.009* | 1.46 (1.00-2.12) | 0.046* | 1.87 (1.23-2.84) | 0.003* |
| Priapism | 2.43 (1.12-5.27) | 0.024* | 2.10 (1.24-3.56) | 0.006* | 1.35 (0.73-2.49) | 0.334 |
| Acute Chest Syndrome | 1.99 (1.12-3.54) | 0.018* | 1.76 (1.10-2.82) | 0.018* | 1.56 (1.03-2.37) | 0.033* |
| Odds ratios (OR) are reported per additional copy of the *BCL11A* TTA⁻ haplotype or per unit increase in the genetic risk score (GRS). Hazard ratios (HR) represent the per-copy effect of *BCL11A* TTA⁻ haplotype on the cumulative risk of complications. All p-values are adjusted for covariates (age, gender, and vaso-occlusive crises (VOCs) per year). | | | | | | |

**Supplementary Table S8:** Logistic and Cox proportional regression analyses of *BCL11A* haplotypes and genetic risk score with SCA clinical complication

**Supplementary table S9:** Association of the genetic risk score (GRS) with SCA clinical complications. Logistic regression analyses assessing the association between the GRS and various SCA clinical complications. Predictor variables include GRS, age (continuous variable), gender (male vs. female), and vaso-occlusive crises (VOCs) episodes *per year*, categorized as >5 vs. 0–5 events.

| **Clinical Complication** | **Variable** | **OR (95% CI)** | ***p-value*** |
| --- | --- | --- | --- |
| Stroke | Genetic Risk Score | 1.28 (1.07-1.55) | 0.008* |
|  | Age | 1.03 (0.99-1.06) | 0.087 |
|  | Gender (M vs F) | 1.76 (0.92-3.41) | 0.089 |
|  | VOCs (>5 vs 0-5) | 0.79 (0.24-2.23) | 0.677 |
| Avascular necrosis | Genetic Risk Score | 1.25 (1.05-1.50) | 0.013* |
|  | Age | 1.07 (1.04-1.10) | <0.001* |
|  | Gender (M vs F) | 2.64 (1.39-5.13) | 0.003* |
|  | VOCs (>5 vs 0-5) | 2.54 (1.09-5.92) | 0.030* |
| Leg Ulcers | Genetic Risk Score | 1.35 (1.14-1.62) | <0.001* |
|  | Age | 1.08 (1.05-1.12) | <0.001* |
|  | Gender (M vs F) | 3.71 (2.05-6.90) | <0.001* |
|  | VOCs (>5 vs 0-5) | 2.03 (0.91-4.61) | 0.086 |
| Priapism | Genetic Risk Score | 1.30 (1.02-1.68) | 0.040* |
|  | Age | 1.03 (0.99-1.07) | 0.186 |
|  | VOCs (>5 vs 0-5) | 1.81 (0.59-6.22) | 0.314 |
| Acute Chest Syndrome | Genetic Risk Score | 1.33 (1.11-1.62) | 0.003* |
|  | Age | 1.02 (0.99-1.05) | 0.160 |
|  | Gender (M vs F) | 2.09 (1.13-3.92) | 0.020* |
|  | VOCs (>5 vs 0-5) | 3.36 (1.55-7.47) | 0.002* |
| Results are reported as odds ratios (OR) with 95% confidence intervals (CI) and corresponding p-values, where OR >1 indicates increased risk, while OR <1 suggests a protective effect for the first category listed. Adjusted p-values are presented. | | | |

**Supplementary Table S10:** Minor allele frequencies of *BCL11A* and *HBS1L-MYB* variants in major ancestral population groups contributing to Brazilian admixture.

| **Gene** | **SNP** | **Minor allele (HbF-boosting)** | **AFR** | **EUR** |
| --- | --- | --- | --- | --- |
| *BCL11A* | rs4671393 | A | 0.27 | 0.17 |
| *BCL11A* | rs1427407 | T | 0.24 | 0.15 |
| *BCL11A* | rs11886868 | C | 0.26 | 0.31 |
| *HBS1L-MYB* | rs9399137 | C | 0.04 | 0.26 |
| AFR, African populations; EUR, European populations.  Allele frequency data were retrieved from the Ensembl genome browser, which aggregates population frequency information from the 1000 Genomes Project. | | | | |


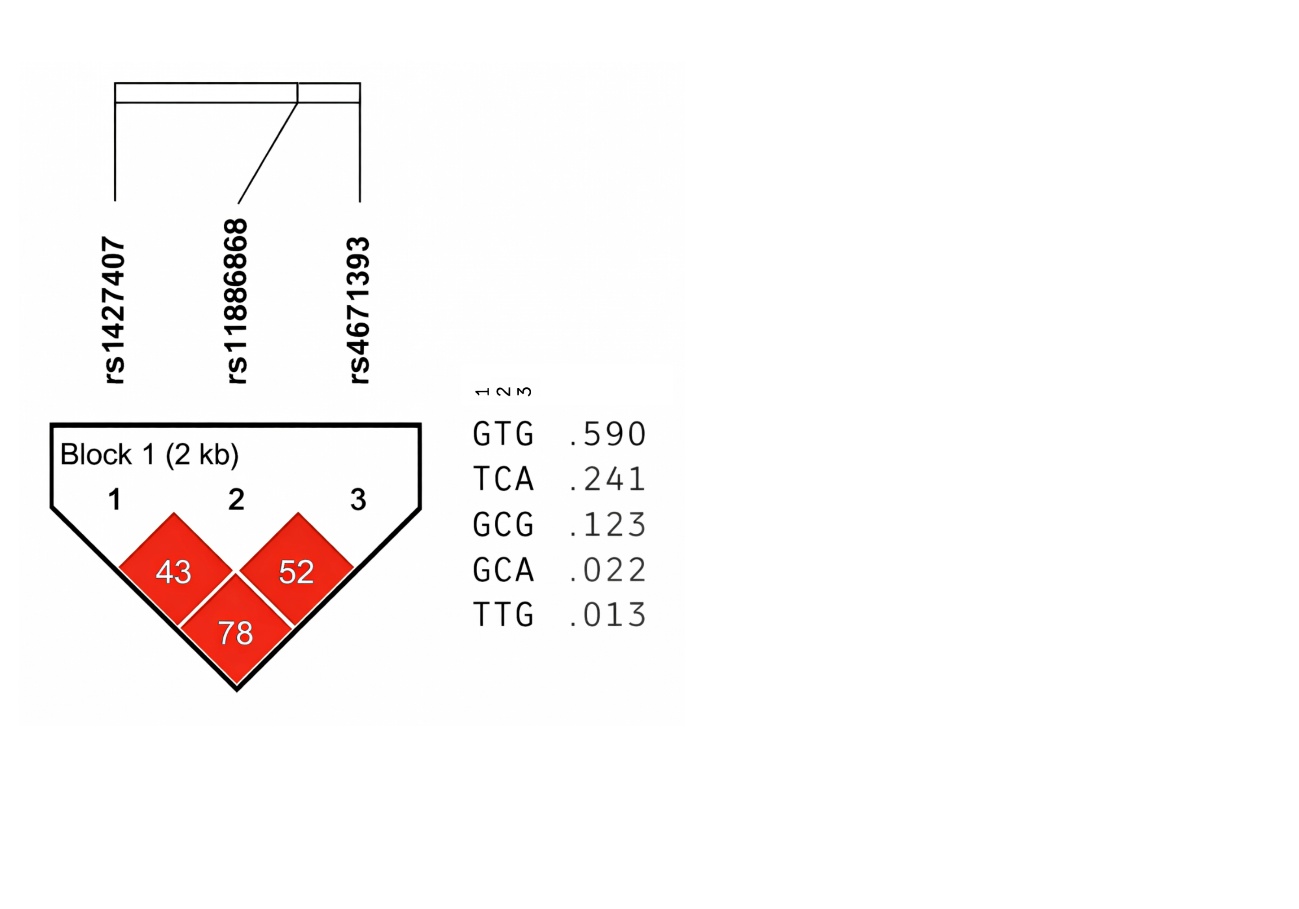


(a)

(b)

**Supplementary figure S1**: Linkage disequilibrium analysis of rs1427407, rs11886868, and rs4671393 of *BCL11A* using the solid spine of LD method. (a) HaploView 4.2 color schemes, white corresponds to D’ < 1 and LOD < 2, shades of pink/red indicate D’ < 1 and LOD ≥ 2, and light blue indicates complete LD with D’ = 1 and LOD < 2. Values inside the rhombuses indicate r^2^. (b) Identified haplotypes and associated frequencies.


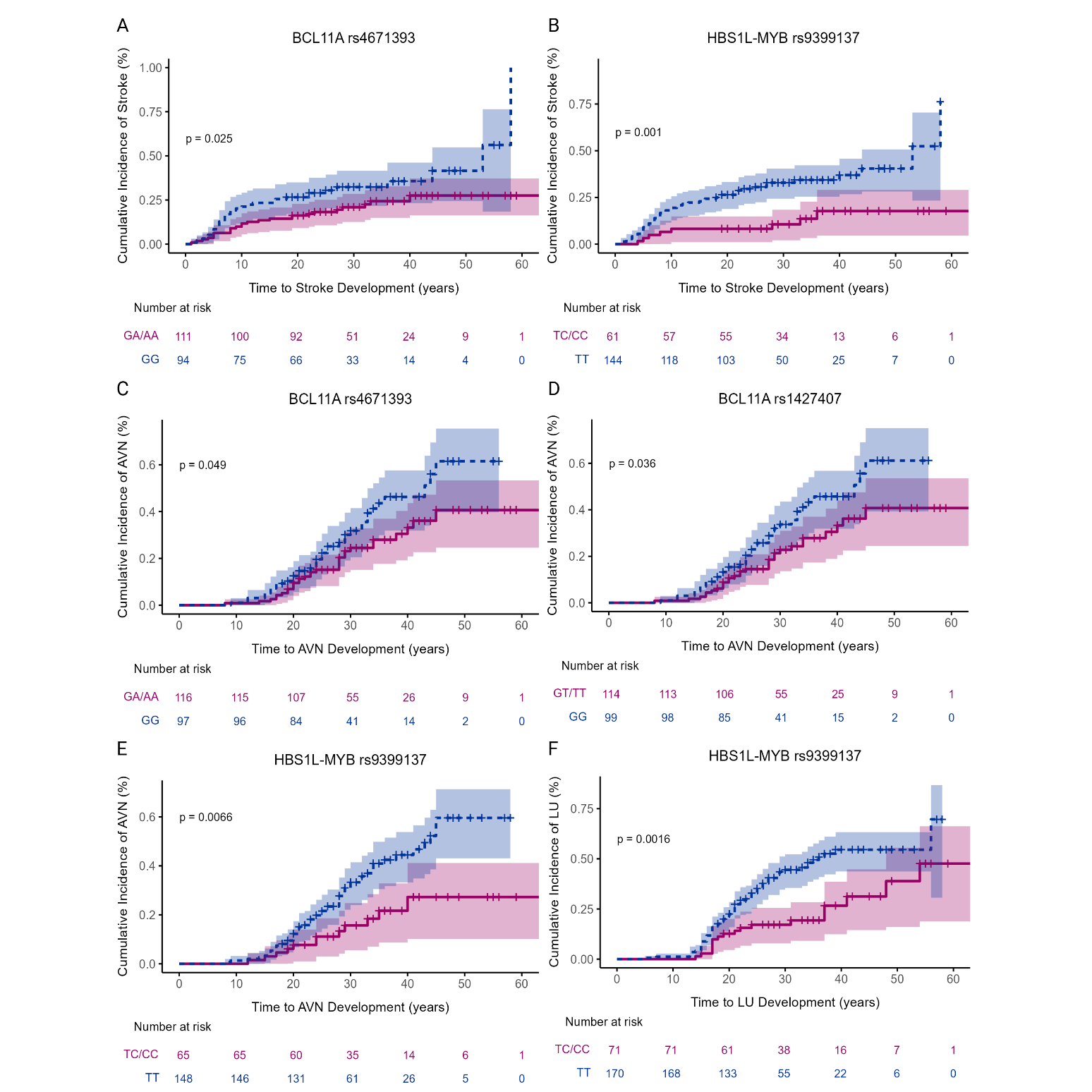


**Supplementary figure S2:** Kaplan-Meier cumulative incidence curves for clinical complications in SCA patients stratified by *BCL11A* and *HBS1L-MYB* genetic variants. (A) Association of *BCL11A* rs4671393 with stroke incidence. (B) Association of *HBS1L-MYB* rs9399137 with stroke incidence. (C) Association of *BCL11A* rs4671393 with avascular necrosis (AVN) incidence. (D) Association of *BCL11A* rs1427407 with AVN incidence. (E) Association of *HBS1L-MYB* rs9399137 with AVN incidence. (F) Association of *HBS1L-MYB* rs9399137 with leg ulcer (LU) incidence. The shaded areas represent 95% confidence intervals, and log-rank p-values indicate the statistical significance of differences between genotype groups.


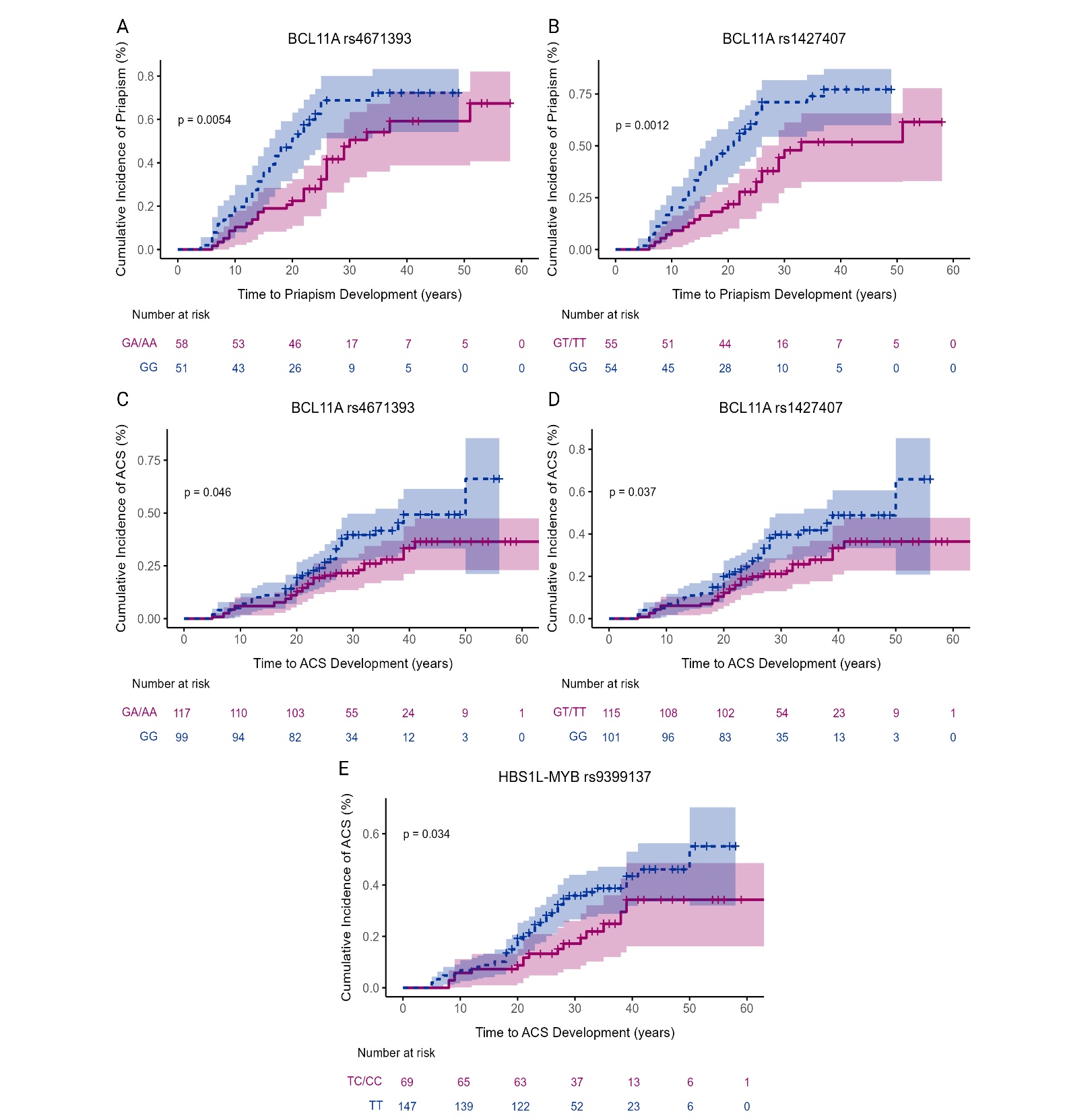


**Supplementary figure S3:** Kaplan-Meier cumulative incidence curves for clinical complications in SCA patients stratified by *BCL11A* and *HBS1L-MYB* genetic variants. (A) Association of *BCL11A* rs4671393 with priapism incidence. (B) Association of *BCL11A* rs1427407 with priapism incidence. (C) Association of *BCL11A* rs4671393 with acute chest syndrome (ACS) incidence. (D) Association of *BCL11A* rs1427407 with ACS incidence. (E) Association of *HBS1L-MYB* rs9399137 with ACS incidence. The shaded areas represent 95% confidence intervals, and log-rank p-values indicate the statistical significance of differences between genotype groups.


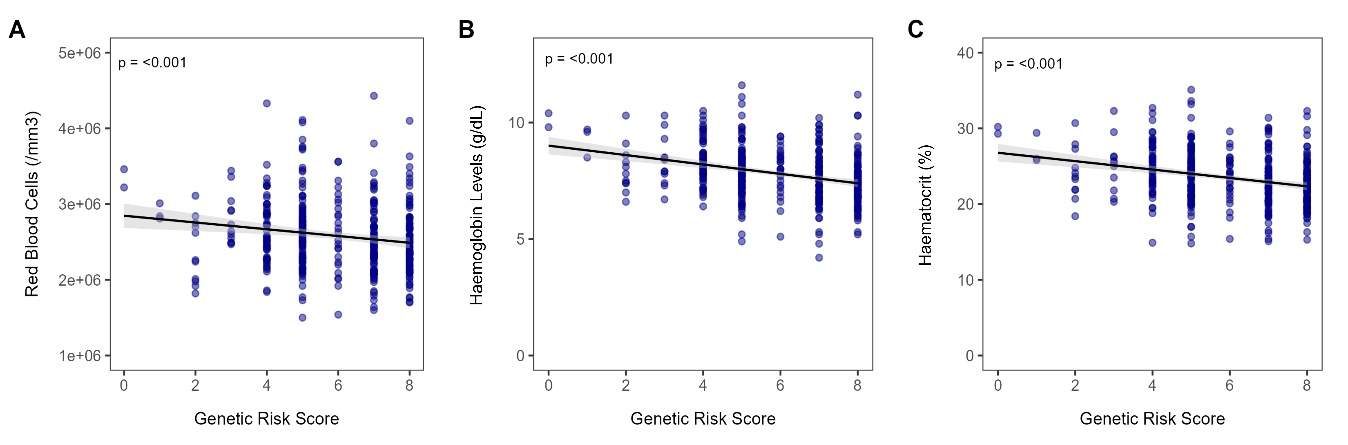


**Supplementary figure S4:** Association of the genetic risk score (GRS) with haematological parameters in sickle cell anaemia (SCA) patients. (A) Association between the GRS and red blood cell count (/mm^3^) in SCA patients. (B) Association between the GRS and haemoglobin levels (g/dL) in SCA patients. (C) Association between the GRS and haematocrit (%) in SCA patients. The black line represents the logistic regression fit with a 95% confidence interval (shaded area).
